# A DNA barcode library of Austrian geometridae (Lepidoptera) reveals high potential for DNA-based species identification

**DOI:** 10.1371/journal.pone.0298025

**Published:** 2024-03-11

**Authors:** Benjamin Schattanek-Wiesmair, Peter Huemer, Christian Wieser, Wolfgang Stark, Axel Hausmann, Stephan Koblmüller, Kristina M. Sefc

**Affiliations:** 1 Tiroler Landesmuseen Betriebsges.m.b.H., Innsbruck, Austria; 2 Institute of Biology, University of Graz, Universitätsplatz, Graz, Austria; 3 Landesmuseum Kärnten, Klagenfurt am Wörthersee, Austria; 4 Ökoplus Umweltforschung und Consulting GmbH, Trübensee, Austria; 5 Zoologische Staatssammlung München, München, Germany; Nanjing Agricultural University, CHINA

## Abstract

Situated in the Eastern section of the European Alps, Austria encompasses a great diversity of different habitat types, ranging from alpine to lowland Pannonian ecosystems, and a correspondingly high level of species diversity, some of which has been addressed in various DNA barcoding projects. Here, we report a DNA barcode library of all the 476 species of Geometridae (Lepidoptera) that have been recorded in Austria. As far as possible, species were sampled from different Austrian regions in order to capture intraspecific genetic variation. In total, 2500 DNA barcode sequences, representing 438 species, were generated in this study. For complete coverage of Austrian geometrid species in the subsequent analyses, the dataset was supplemented with DNA barcodes from specimens of non-Austrian origin. Species delimitations by ASAP, BIN and bPTP methods yielded 465, 510 and 948 molecular operational taxonomic units, respectively. Congruency of BIN and ASAP partitions with morphospecies assignments was reasonably high (85% of morphospecies in unique partitions), whereas bPTP appeared to overestimate the number of taxonomic units. The study furthermore identified taxonomically relevant cases of morphospecies splitting and sharing in the molecular partitions. We conclude that DNA barcoding and sequence analysis revealed a high potential for accurate DNA-based identification of the Austrian Geometridae species. Additionally, the study provides an updated checklist of the geometrid moths of Austria.

## Introduction

**Fig 1:**
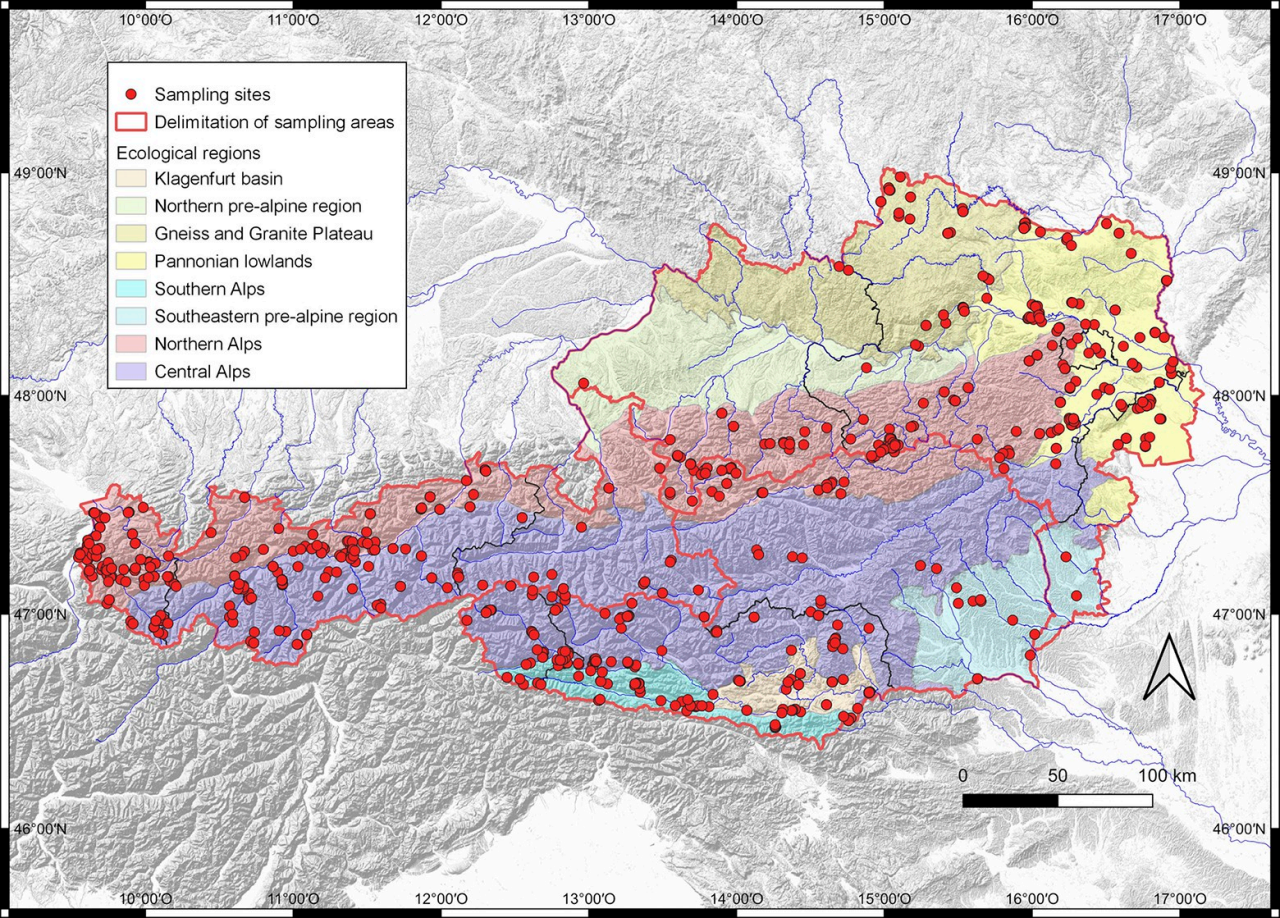
Map of sampling locations.

Austria is a landlocked Central European country in the intersection of three biogeographic regions [1]. Situated in the Eastern section of the European Alps, a large part of the country’s approximately 84,000 square kilometers is assigned to the alpine biogeographic region, with elevations ranging up to 3798 MSL in the Großglockner mountain massive. The lowlands belong to the continental region, and Pannonian influences are evident in the landscape, fauna and flora of the northeastern part of the country, where the lowest point of Austria lies at 114 MSL. On a more regional scale, heterogeneity in geological, geomorphological and climatic characteristics is reflected in the classification of eight distinct ecological regions within Austria ([2]; Fig 1).

Sampling sites of Austrian specimens are marked by red dots, and red lines delimit the three sampling areas (North-Eastern Austria, Southern Austria, Western Austria). The map was drawn using the following data: Hillshades, source HeiGIT, used with permission from HeiGIT (Heidelberg Institute for Geoinformation Technology). Administrative borders, source Eurostat (https://ec.europa.eu/eurostat/web/gisco/geodata/reference-data/administrative-units-statistical-units/nuts), download date: February 15, 2023, copyright information: © EuroGeographics, © TurkStat. Source: European Commission–Eurostat/GISCO. Ecoregions, modified from GIS data (https://www.data.gv.at/katalog/dataset/0cf499e1-ac26-47d6-8f8f-afe03d0cc5c7) provided by Umweltbundesamt GmbH under a CC BY 4.0 license (https://creativecommons.org/licenses/by/4.0/).

The exceptionally high ecosystem diversity of Austria is mirrored in high levels of species diversity, with hotpots of regional endemism in the alpine region [3]. For instance, with more than 4070 species of butterflies and moths [4], the lepidopteran species richness of Austria exceeds that of all other Central European countries [5]. In total, over 54,000 animal species have been reported to occur in Austria [6]. However, while species counts have been on the rise in recent decades (in part due to intensified research efforts), the decline of overall biomass and the increasing number of endangered species testify to the pressure experienced by the biodiversity of this biogeographically richly structured area [6]. The stock-taking of Austrian species has recently gained momentum through various DNA barcoding projects, covering a wide range of animal taxa from fish [7], amphibians and reptiles [8], acanthocephalans (e.g. [9, 10]), proturans [11, 12] to various insect taxa (e.g. Odonata:[13]; Orthoptera: [14]; Boreidae: [15]; Ceratopogonidae: [16]; mosquitos: [17, 18]. With respect to Lepidoptera, DNA barcoding has been almost completed for Austrian butterfly species (superfamily Papilionoidea, [19]) and noctuid moths (superfamily Noctuoidea, [20]), and barcoding of the European species of microlepidopteran Gelechiidae and the leaf mining Gracillaridae included most of the species recorded from Austria [21, 22]. Moreover, the lepidopteran fauna of individual federal states has been comprehensively covered [23, 24].

A highly diverse family of Lepidoptera are the Geometridae with approx. 24,000 species described worldwide [25–27] and divided in eight [28, 29] or nine subfamilies [26]. While the largest diversity of geometrid moths is found in tropical regions, six or seven of the subfamilies occur in Europe (Larentiinae, Geometrinae, Ennominae. Sterrhinae, Orthostixinae, Desmobathrinae and Archiearinae; [30], with Orthostixinae possibly requiring integration in Desmobathrinae [26]. Over two decades, the extensive taxonomic research on the approximately 1,000 European species of geometrid moths has been compiled in the book series “The Geometrid Moths of Europe” [31–36]. Recently, the taxonomic work on geometrid moths has been complemented by DNA barcoding efforts [37, 38], occasionally giving rise to the discovery of new species [32, 39–42]. Almost half of the European geometrid species are also found in Austria. The most recent checklist of the Austrian Lepidoptera fauna lists 473 Geometridae species [4], and several small taxonomic changes [32, 43–45] have since then raised the national species count to 476 (see updated checklist of Austrian geometrid moths in S1 Table). The biological and morphological diversity within geometrid moths includes the evolution of behavioural strategies such as day activity as well as morphological adaptations to cold and windy climate conditions reflected in winglessness or brachyptery in the females of some species. These adaptions allow Geometridae to occupy nearly all Austrian habitat types from the lowlands to the subnival zone. In particular, structured and fragmented alpine habitats likely promote diversification and regional endemism. Two endemic species, *Elophos zirbitzensis* (Pieszczek, 1902) and *Sciadia innuptaria* (Herrich-Schäffer, 1852), as well as several subendemic species and endemic subspecies occur in the Austrian alpine region [3, 4]. These and other alpine species and subspecies are restricted to alpine habitats and threatened by climate warming [46]. A national red list for geometrid moths is lacking, but red lists exist for some of the Austrian provinces and report alarmingly large numbers of endangered species, including 139 endangered species in Vorarlberg (corresponding to 38.9% of geometrid species known in the province) [47], 132 species endangered in Salzburg [48], 139 (36.0%) species in Upper Austria [49] and 196 (49.5%) species in Carinthia [50]. Furthermore, two species listed in annex 2 and 4 of the European Habitat Directive occur in the Eastern Austrian provinces Lower Austria and Burgenland (*Chondrosoma fiduciaria* Anker, 1854 and *Lignyoptera fumidaria* (Hübner, 1825)).

The present study assembled DNA barcodes for all 476 Austrian geometrid species. A particular effort was made to sample with broad geographic coverage in order to capture intraspecific genetic variation. We use these data to evaluate the potential for DNA-based species identification, which is crucial, for instance, to meta-barcoding applications for monitoring purposes [51]. We also discuss causes and potential taxonomic consequences of incongruencies between current classification and genetic patterns (BIN-sharing, BIN-splitting).

## Material and methods

### Sampling

In this study, we compiled a dataset comprising DNA barcode sequences of all of the 476 species of Geometridae occurring in Austria. Of these, 438 species were represented by at least one specimen collected in Austrian, while the remaining 38 species were represented by specimens of non-Austrian origin (see details below). Specimens were collected during faunistic research or were acquired from existing collections. Twenty-eight research collections contributed to this study, with most specimens originating from Tiroler Landesmuseum Ferdinandeum (Innsbruck) (1333 specimens), the research collection of Wolfgang Stark (474 specimens), Landesmuseum Kärnten (Klagenfurt) (376 specimens) and inatura (Dornbirn) (142 specimens). Our sampling was designed with the aim to maximise geographic coverage. Therefore, we divided Austria in three areas: (1) North-Eastern Austria, encompassing the provinces Burgenland, Vienna, Lower Austria and Upper Austria, and including much of the northern pre-alpine region, the Pannonian lowlands and the Gneiss and Granite Plateau; (2) Southern Austria (Styria, Carinthia and East Tyrol), consisting of alpine and pre-alpine regions; and (3) Western Austria (Salzburg, North Tyrol, Vorarlberg), mostly belonging to alpine ecological region. Sample coverage of the three areas was achieved for 272 species, and 79 species were sampled from two areas. The remaining species either occur in only one of the areas [4], or sample collection from the other areas failed although the species has been recorded there. In any case, we aimed at sampling at least three specimens per species. However, for 38 species reported to occur in Austria, no DNA barcode sequences could be generated due to lack of Austrian samples or insufficient quality for successful sequencing. In order to represent these species in the analyses, we retrieved one COI sequence per species from the Barcode of Life Data System (BOLD; www.boldsystems.org [52]), selecting database entries from the geographically nearest sampling sites (mostly from Germany, but ranging from the Iberian Peninsula to Finland; S1 Table).

### Specimen identification

Identification of the morphospecies was based on the “Geometrid moths Europe” series [31–36]. In species where identification based on external morphology was not possible, a dissection of the genitalia was performed. After examination, genitalia were stored in glycerine in a vial pinned underneath the specimen. For comparisons of genitalia morphology between specimens assigned to different BINs within *Gagitodes sagittata* and within *Tephronia sepiaria*, genitalia were fixed on slides following the method of [53]. Photographs of the genitalia preparations were taken with a Panasonic Lumix GH4 mounted on an Olympus BH-2 microscope. The pictures were stacked with Helicon Focus 8 software (HeliconSoft, Ukraine). Adobe Photoshop CS6 and GIMP 2.10.8 (https://www.gimp.org/) were used to add scale bars, assemble photographs of female genitalia and remove background noise.

### DNA barcoding and data analysis

Dry legs of 2734 specimens were sent to the Canadian Centre for DNA Barcoding (CCDB, Biodiversity Institute of Ontario, University of Guelph) for sequencing the DNA barcode region of the mitochondrial COI gene (cytochrome c oxidase subunit 1). The DNA barcode sequences were generated using a standard high-throughput protocol [54] using primers LepF1 and LepF2 [55]. Resulting sequences were checked for DNA barcode compliance in the BOLD system, and sequences > 500 bp that met these criteria were retained for further analyses. Specimen collection data and images are publicly available in the BOLD dataset “DS-LEATGEOM Lepidoptera (Geometridae) of Austria”, and DNA sequences were also deposited in Genbank (Genbank accession numbers in S1 Text).

### Data analysis

For each species, the nearest neighbor Kimura two-parameter (K2P) distances (min.NN) as well as mean and maximum intraspecific K2P distances (for species with > 1 sample) were calculated on the BOLD system v. 4.0 using the Barcode Gap Analysis tool, with pairwise deletion of missing/ambiguous characters. Nearest neighbor distances were calculated between the focal species and the most similar COI sequence of the nearest neighbor species in the dataset.

Sequences were assigned Barcode Index Numbers (BINs; [56]). BINs are based on an algorithm that clusters all high-quality sequences from BOLD into operational taxonomic units, regardless of their previous taxonomic assignment. We recorded the number of BINs per species, that were detected in the Austrian specimens. For each of the 476 Austrian geometrid species, we also determined the number of BINs per species in datasets including all European specimens (excluding Russian and Turkish specimens). These data were derived from BOLD in March 2022 and were used to plot the number of intra-specific BINs detected in Austria against the number of intra-specific BINs of the same species present across Europe.

Additionally, molecular operational taxonomic unit (MOTU) delimitation was carried out with ASAP [57] and bPTP [58]. The assignment of species to subfamilies follows the systematic checklist of European Geometridae [30]. ASAP is a distance-based method to partition sequence datasets into MOTUs and was calculated on the webserver (https://bioinfo.mnhn.fr/abi/public/asap/) using the K2P model with ts/tv set to 2. Finally, we used a Bayesian implementation of the PTP model for species delimitation (bPTP), which is based on a rooted phylogenetic tree and predicts branching events and speciation based on the numbers of substitutions. The maximum likelihood (ML) trees to be used as input files for the bPTP analysis were constructed via the Phylosuite v.1.2.2 platform [59]. The following plugins were used: The alignment was performed with MAFFT v7.313 [60], the best fitting model was calculated with Modelfinder (IQ-TREE v.1.6.8) [61] and the maximum likelihood tree was inferred with IQ-TREE v.1.6.8 [62] with the setup of an automatic substitution model and 20000 ultrafast bootstrap replicates.

In addition to the output retrieved from BOLD, summary statistics were calculated and plotted in R v. 4.1.3 (R Development Core Team 2022). The bPTP analysis split numerous species into several MOTUs, and the relationship between numbers of bPTP-MOTUs per species and sample size was examined in a generalized linear model with a negative binomial error distribution using the R package glmmTMB [63].

Phylogenetic relationships within individual taxonomic groups were represented by Neighbor Joining trees based on K2P distances (with pairwise deletion of missing/ambiguous characters) constructed using MEGA X [64]. The trees were visualized with FigTree v1.4.4 [65] and GIMP 2.10.8 [66].

## Results

### DNA barcode sequence generation

In this study, 2567 sequences (>500 bp) of specimens of Austrian origin were generated, covering 438 species (92% of the Austrian geometrid species). Of these, 2500 sequences fulfilled the criteria of DNA barcode compliance and 2525 sequences were assigned a Barcode Index Number (BIN). Sequencing failed entirely for 118 specimens, and 49 sequences of lengths < 500 bp were excluded from further analyses.

Across all species (n = 476, including the 38 species that were represented by non-Austrian specimens), sample sizes ranged from 1–24 specimens per species (median = 5.0). For 365 species, we achieved our goal to obtain three or more DNA barcode sequences, while 44 species were represented by two DNA barcode sequences and 67 species were represented by a single DNA barcode sequence.

### Intra- and interspecific genetic distances

**Fig 2:**
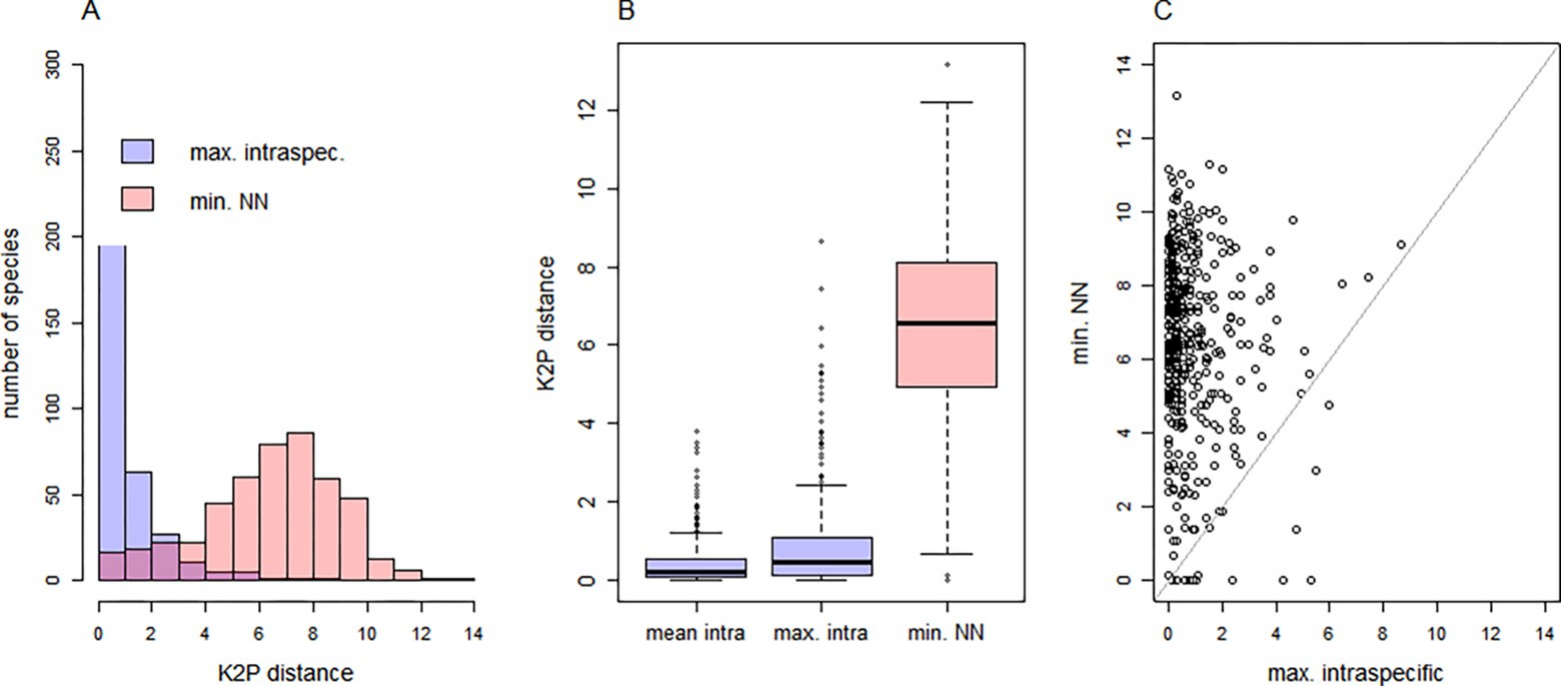
Intraspecific distances and distances to nearest neighbor. (A) Histograms of maximum intraspecific distance (max. intraspec.) and minimum distances to the nearest species (min.NN) for the investigated species. (B) Boxplots of mean intraspecific distances (mean intra), maximum intraspecific distances (max. intra) and minimum distances to nearest neighbor species (min. NN). (C) Scatterplot of maximum intraspecific against minimum interspecific (min. NN) distances illustrating the barcoding gap for species plotted above the diagonal line. All distances were calculated using the K2P model.

In species with two or more DNA barcode sequences (n = 409 species), mean intraspecific K2P distances ranged from 0% to 3.81% (average across species = 0.42%; median = 0.21%), and maximum intraspecific K2P distances ranged from 0% to 8.7% (average across species = 0.88%; median = 0.46%; Fig 2A and 2B).

Across all species (n = 476 species), minimum interspecific distances to the nearest neighbor (min.NN) ranged from 0% to 13.15% (average across species = 6.37%; median = 6.57%; Fig 2A and 2B), and fell below 2% for 34 species (7.1% of 476 species; S1 Table).

For the vast majority of species (97.5%), min.NN exceeded their maximum intraspecific distance (Fig 2C).

### Species delimitation

**Fig 3:**
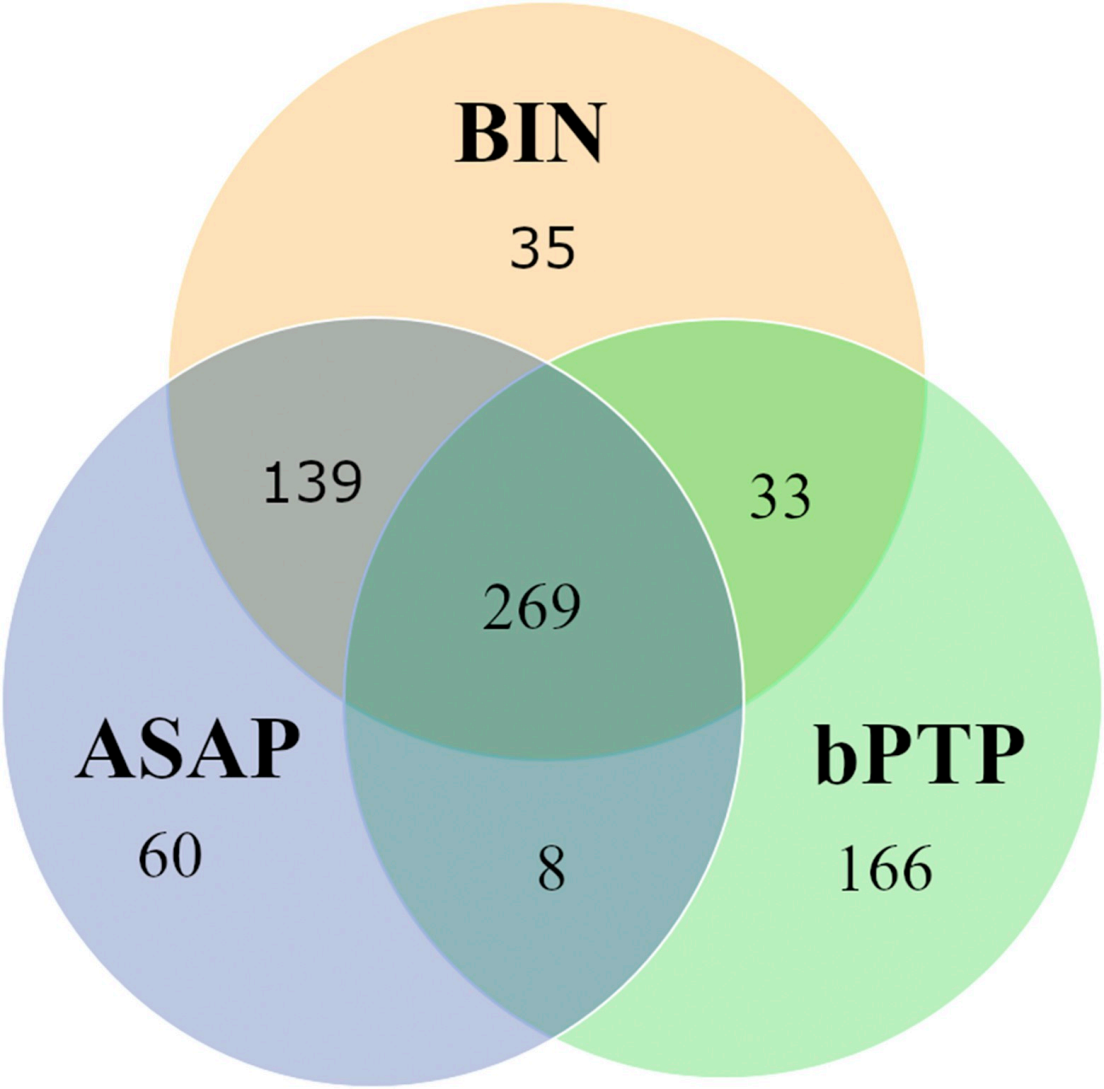
Venn diagram illustrating congruence and incongruence of MOTU assignment between the three methods used in this study. Number of species, for which MOTU assignments were consistent (overlapping areas) or inconsistent across species delimitation methods.

Species delimitation by means of ASAP, the BIN system and bPTP yielded 465, 510 and 948 molecular operational taxonomic units (MOTUs) across the full dataset covering 476 morphospecies. BIN and ASAP yielded identical MOTU assignments for 417 morphospecies, 269 of which were also retrieved by bPTP (Fig 3). In contrast, for 166 species, their bPTP-based MOTU assignments were not supported by the other algorithms (Fig 3).

**Fig 4:**
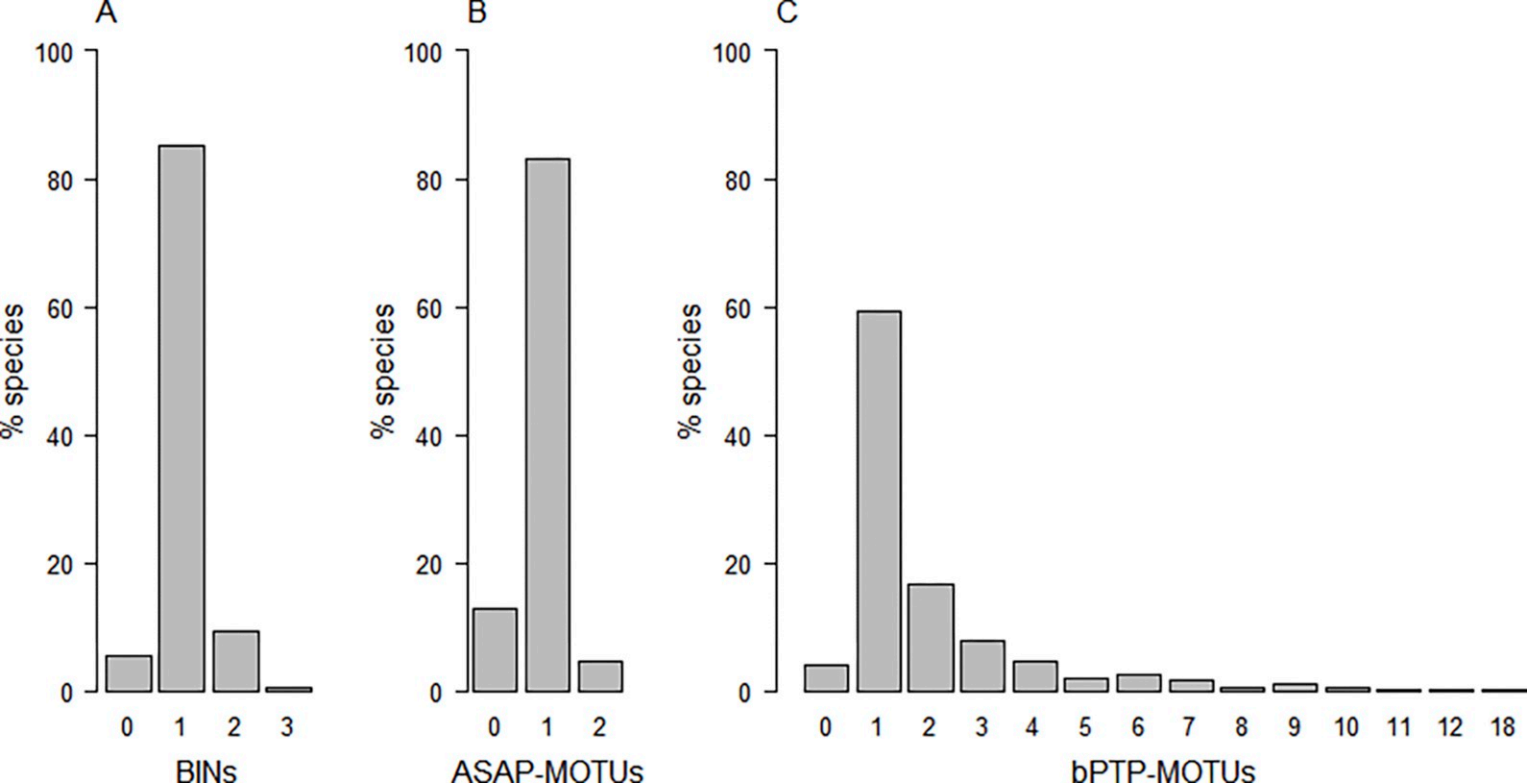
Congruence between MOTU assignments and morphospecies classification. The barplots show the percentage of species that share MOTUs with another species (category 0 = MOTU sharing), are assigned to their unique individual MOTU (category 1 = congruence with morphospecies), or are split into multiple MOTUS (categories 2 and higher = MOTU splitting). (A) BIN assignments; (B) MOTUs as defined by ASAP; (C) MOTUs as defined by bPTP.

Congruency between morphospecies and BIN assignment was high, with unique BINs assigned to 406 (85.3%) of the 476 morphospecies. 27 morphospecies shared BINs with another morphospecies (see Table 1), 47 morphospecies were split into two BINs, and two morphospecies (*Eupithecia icterata* and *Eupithecia satyrata*) were split into three BINs (Fig 4). Four morphospecies showed both BIN-splitting and BIN-sharing (*Elophos caelibaria*, *Rheumaptera subhastata*, *Sciadia tenebraria*, *Sciadia zelleraria*; S1 Table). Broken down by subfamily (Table 2, S2 Table), congruence between BINs and morphospecies assignments was weaker in the species-poor subfamilies Geometrinae and Archiearinae than in the other three, species-rich subfamilies.

**Table 1:** BIN-sharing between morphospecies.

Species (sample size)	Min. NN (K2P)	Samples cluster by species in phylogenetic tree	MOTU shared		Differentiation	Comments
			ASAP	bPTP		
*Lycia alpina* (n = 5) *Lycia zonaria* (n = 3)	0	no	yes	yes	wing coloration and wing shape, habitat preferences	Hybridization reported [32,67]. The same BIN is shared also by another (non-Austrian) species, *L*. *graecarius*.
*Lycia isabellae* (n = 1) *Lycia pomonaria* (n = 1)	1.26	no^a^	yes^b^	no	wing coloration and wing shape, larval host plants, habitat preferences	Hybridization reported [32,67]
*Perizoma affinitata* (n = 6) *Perizoma hydrata* (n = 7)	0.15	no	yes^c^	Yes	wing coloration, male and female genitalia	Low interspecific divergence also in [38]
*Cyclophora punctaria* (n = 8) *Cyclophora quercimontaria* (n = 1) *Cyclophora suppunctaria* (n = 1)	0–0.15	no ^a^	yes	yes	male and female genitalia	Hybridization reported. Species barriers are generally low in the genus *Cyclophora* and natural hybrids were found repeatedly [34,68].
*Thera cembrae* (n = 20) *Thera obeliscata* (n = 10)	0	no	yes	yes	host plants	*T*. *cembrae* is differently interpreted: as valid species but possibly conspecific with *T*. *obeliscata* [36,69]; or as subspecies of *T*. *variata* [70], which is refuted by the current sequence data (Fig 5)
*Sciadia zelleraria* (n = 8) *Sciadia tenebraria* (n = 10) *Sciadia innuptaria* (n = 4)	0	no	yes	yes	wing coloration and wing shape	Hybridization in the genus *Sciadia* is common[32].
*Elophos caelibaria* (n = 14) *Sciadia slovenica* (n = 1)	0	no ^a^	yes	yes	wing coloration and wing shape	Hybridization in the genus *Sciadia* is common [32] and may encompass its sister genus *Elophos*
*Chlorissa cloraria* (n = 7) *Chlorissa viridata* (n = 4)	0	no	yes	yes	ontroversial: differentiation by forewing costa and in male genitalia [35,70], but overlapping traits at least in some geographic regions reported	Potentially conspecific: Morphological distinction unclear, identical DNA barcodes shared across species
*Thera britannica* (n = 9) *Thera variata* (n = 9) *Thera vetustata* (n = 7)	1.0–2.0	yes	yes	no	wing coloration, shape of male antenna	
*Boudinotiana notha* (n = 4) *Boudinotiana puella* (n = 2)	1.4	yes	no	yes	wing coloration and wing shape	
*Chloroclysta siterata* (n = 7) *Chloroclysta miata* (n = 7)	1.4	yes	yes	no	wing coloration and wing shape	
*Rheumaptera hastata* (n = 22) *Rheumaptera subhastata* (n = 5)	1.39	yes	yes	no	male genitalia	

The table lists cases of BIN-sharing between morphospecies, along with sample size per species and the minimum Kimura-2-parameter genetic distance between the involved species (Min. NN K2P). Min. NN distances of 0 indicate identical DNA sequences, i.e. cases of DNA barcode sharing between species. We also indicate whether sequences cluster by species in phylogenetic reconstructions, in which cases DNA-based species discrimination is possible despite BIN sharing; and whether MOTU sharing was also observed in ASAP and bPTP analyses. Finally, we report phenotypic differences between BIN-sharing species and, when possible, offer explanations for the observed BIN sharing.

^a^only 1 sample for one or both species.

^b^together with *L*. *hirtaria*.

^c^together with *Perizoma lugdunaria*.

**Fig 5:**
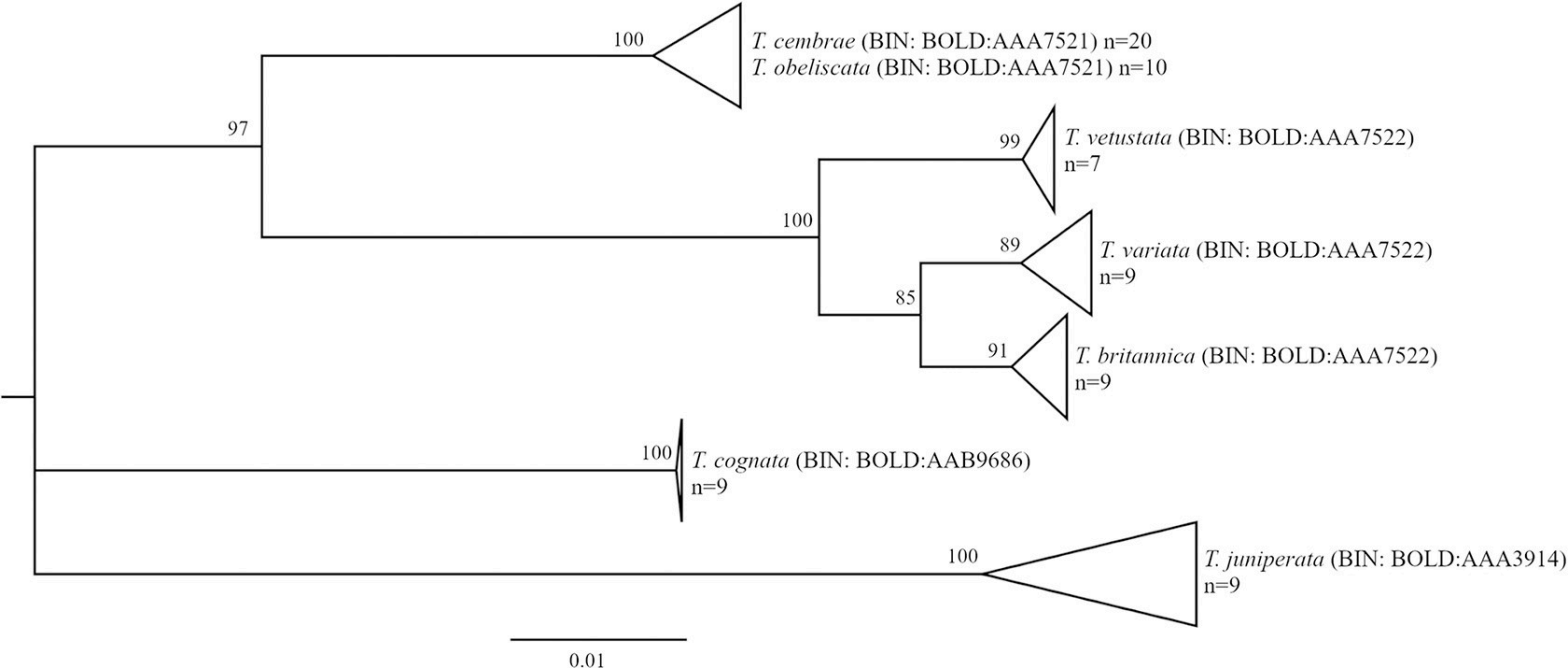
Phylogenetic relationships among species of the genus *Thera*. Neighbor Joining trees based on K2P distances (with pairwise deletion of missing/ambiguous characters), n = sample size; all samples are of Austrian origin.

**Table 2:** Morphospecies and MOTUs of Austrian Geometridae.

		BIN			ASAP			bPTP		
taxonomic group	no. of species	unique	share	split	unique	share	split	unique	share	split
Geometridae	476	406	27	47	396	61	22	282	20	181
By subfamily:										
Geometrinae	13	10	2	1	9	4	0	7	2	6
Archiearinae	3	0	2	1	3	0	0	1	2	0
Ennominae	146	122	9	18	123	16	9	91	7	51
Larentiinae	248	216	11	22	201	38	10	141	6	103
Sterrhinae	66	58	3	5	60	3	3	42	3	21

The table reports the number of Geometridae morphospecies (no. of species) recorded in Austria, and the number of morphospecies assigned unique BINs (unique), the number of morphospecies sharing MOTUs with other morphospecies (share) and the number of morphospecies split into multiple MOTUs (split), for each of the three MOTU delimitation methods. Since both MOTU splitting and sharing occurred in some morphospecies, the sums of the species counts across “unique”, “share” and “split” may exceed the number of species.

**Fig 6:**
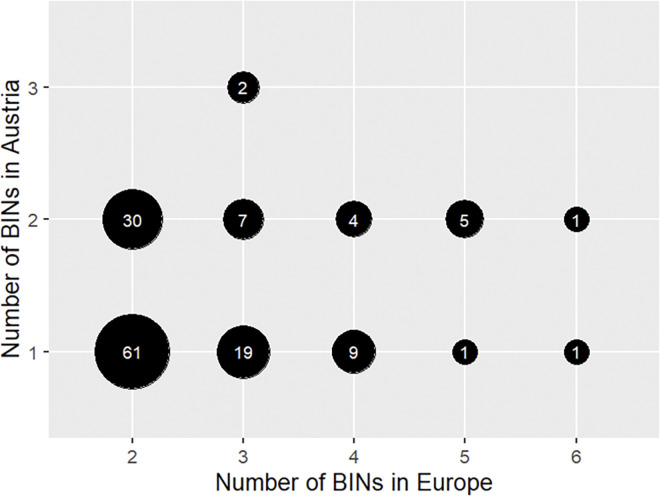
Number of BINs per species detected in Austria, plotted against the number of BINs in the same species across its European distribution. Circle size corresponds to the number of species with identical values; the species count is also reported within each circle.

Across Europe, 140 (i.e., 30%) of the here investigated species split into multiple BINs, with up to six BINs per morphospecies. In Austria, 91 of these species are represented with a single BIN each, and 47 species are each represented with two BINs (Fig 6; S1 Table). Species subjected to BIN splitting, in which maximum intraspecific distance exceeded 3% [71], are listed in Table 3. Most of these BIN splits were supported by ASAP, and even higher numbers of MOTUs per morphospecies were suggested by bPTP (Table 3).

**Table 3:** Morphospecies splitting.

Species	Sample size	Max. intra. K2P	BINs Austria	Additional BINs in Europe	ASAP-MOTUs	bPTP-MOTUs	Distribution	Comments (e.g., geographic distribution and frequencies of BINs in BOLD database)
*Electrophaes corylata*	5	8.65	BOLD:AAZ5334		2	2	overlapping	Both BINs are widespread across Europe.
			BOLD:AAC3785					
*Hypomecis punctinalis*	8	7.43	BOLD:AAB1058	BOLD:AAB1059	2	2	overlapping	High level of DNA polymorphism within BINs [32].
			BOLD:ACA2461					
*Ectropis crepuscularia*	7	6.45	BOLD:AAA2076 BOLD:ACE6053		2	7	overlapping	Both BINs are widespread across Europe.
*Alcis repandata*	8	5.96	BOLD:AAA8484 BOLD:AAA8482		2	3	overlapping	Both BINs are widespread across Europe. BOLD:AAA8482 is shared with *A*. *extinctaria* from Altai mountains.
*Horisme tersata*	19	5.48	BOLD:AAC5135		1^a^	5	overlapping	BOLD:AAC5135 is widespread across Europe; BOLD:ABW1783 is shared between one Austrian specimen and specimens from Chinese origin
			BOLD:ABW1783					
*Sciadia zelleraria*	19	5.31	BOLD:AAB4957	BOLD:ACE3560	2	2	overlapping	Hybridization among *Sciadia* species is common [32] and may be the origin of heterospecific haplotypes. Note that some haplotypes of *S*. *zellaria* are shared with *S*. *tenebraria* and *S*. *innuptaria* (Table 1).
			BOLD:AAD2991	BOLD:ABX0095				
				BOLD:ACJ3674				
*Coenotephria salicata*	16	5.27	BOLD:AAB9029 BOLD:AAC8889		2	9	overlapping	BOLD:AAB9029 is widely distributed across Europe, BOLD:AAC8889 is restricted to Germany, Austria and England
*Pasiphila rectangulata*	14	5.08	BOLD:AAA3076 BOLD:AAA3075		2	5	overlapping	BOLD:AAA3075 consists of European and Northern American samples, BOLD:AAA3076 only of European samples.
*Eupithecia satyrata*	21	4.94	BOLD:ACT7251		2	6	overlapping	Each of the three BINs is distributed widely across Europe; BOLD:ACT7251 occurs at high frequency.
			BOLD:AAA4219					
			BOLD:AAA5442					
*Rheumaptera subhastata*	7	4.75	BOLD:AAA5436 BOLD:AAA5435		2^b^	4	overlapping	BOLD:AAA5435 is more closely related to its sister species *R*. *hastata* (1.43% K2P) than to the conspecific BIN BOLD:AAA5436.
*Gagitodes sagittata*	3	4.62	BOLD:AAD8985		2	3	overlapping	BOLD:AAD8985 found in Finland, Northern Italy and in Austria (Vorarlberg and Tyrol), BOLD:AAD8984 in Germany (Bavaria) and Austria (Styria; Fig 7). No differences in genital morphology were detected (n = 1 male and 1 female for each BIN).
			BOLD:AAD8984					
*Sciadia tenebraria*	10	4.27	BOLD:ACE3562	BOLD:AAB4956	2	2	overlapping	Hybridization among *Sciadia* species is common [32] and may be the origin of heterospecific haplotypes. Note that some haplotypes of *S*. *tenebraria* are shared with *S*. *zellaria* and *S*. *innuptaria* (Table 1).
			BOLD:AAB4957	BOLD:ACE3563				
				BOLD:AAC5403				
				BOLD:ACE3561				
*Eupithecia plumbeolata*	12	4.04	BOLD:AAB8936 BOLD:AAB8937	BOLD:ACF3745	2	2	overlapping	BOLD:AAB8936, BOLD:AAB 8937 were found across Europe. BOLD:AAB8937 is shared with the Asian species *E*. *nomogrammata*. BOLD:ACF3745 is known from Finland and Turkey.
*Agriopis bajaria*	5	3.81	BOLD:AEE3670	BOLD:AAC3209	2	5	non-overlapping	High level of DNA polymorphism within BINs [32]
			BOLD:AAC3211	BOLD:AAZ7585				
*Elophos operaria*	3	3.80	BOLD:ADO6177		2	2	non-overlapping	BOLD:ADO6177 represents the nominotypical subspecies; the specimen in BOLD:AEE3881 was collected from the locus typicus (Zirbitzkogel) of the subspecies *E*. *operaria hoefneri*. [32] found no morphological evidence for subspecies recognition.
			BOLD:AEE3881					
*Epirrita autumnata*	10	3.75	BOLD:AAA5906	BOLD:ACE7803	1	2	overlapping	BOLD:AAA5907 is shared with *E*. *filigrammaria* (an endemite of Great Britain); possibly due to introgression [36].
			BOLD:AAA5907	BOLD:AAA5909				
				BOLD:ABY8748				
*Epirrhoe galiata*	9	3.64	BOLD:ACE4142		1	3	overlapping	Both BINs are widespread across Europe.
			BOLD:ACE4676					
*Idaea seriata*	13	3.53	BOLD:AAA9645 BOLD:ACF4900	BOLD:ABZ4137 BOLD:ABY6334	2	2	overlapping	BOLD:AAA9645 is shared with *I*. *minuscularia* (South-Western Europe). BOLD:ABZ4137 and BOLD:ABY6334 are restricted to southern Italy.
*Tephronia sepiaria*	4	3.47	BOLD:AAD2603	BOLD:ADK9120	2	3	non-overlapping	BOLD:AAD2603 is distributed in Western Europe. BOLD:ABV4483 is shared with specimens from Turkey and Greece (Fig 8); subspecies status of this BIN has been taken into consideration [32]. Minor differences in genital morphology were detected (Fig 10; n = 1 male and 1 female for each BIN).
			BOLD:ABV4483					
*Eulithis populata*	6	3.47	BOLD:ADF0720 BOLD:ABZ1837		1	2	overlapping	BOLD:ABZ1837 is widespread and frequent (40 DNA barcode sequences on BOLD); BOLD:ADF0720 currently contains only one Austrian sequence.
*Eupithecia subfuscata*	21	3.40	BOLD:ABY4251	BOLD:ABY4252	1	6	overlapping	BOLD:ABY4251 and BOLD:ACE8007 are widespread across Europe. BOLD:ABY4252, BOLD:ABW4471 are restricted to the Netherlands, with one sequence each.
			BOLD:ACE8007	BOLD:ABW4471				
*Xanthorhoe spadicearia*	8	3.23	BOLD:AAB7980 BOLD:AAB7981		1	2	overlapping	BOLD:AAB7980 and BOLD:AAB7981 are widespread across Europe.
*Lomaspilis marginata*	9	3.16	BOLD:AAB5300		1	1	overlapping	BOLD:AAB5300 is frequent and widespread across Europe; BOLD:ABZ2599 has been found Austria and Poland
			BOLD:ABZ2599					

The table provides information on morphospecies that were split into multiple BINs with K2P distance > 3%. For each species, sample sizes and maximum intraspecific K2P genetic distances (Max. intra. K2P) are reported, and intraspecific BINs detected in Austria as well as additional BINs detected elsewhere in Europe are identified. Furthermore, we report the number of intraspecific ASAP- and bPTP-MOTUs and indicate whether the distributions of intraspecific BINs overlap geographically. In the last column, we compiled information related to the BIN structure in the species.

^a^ together with *H*. *radicaria*.

^b^ one ASAP MOTU shared with *R*. *hastata*.

**Fig 7:**
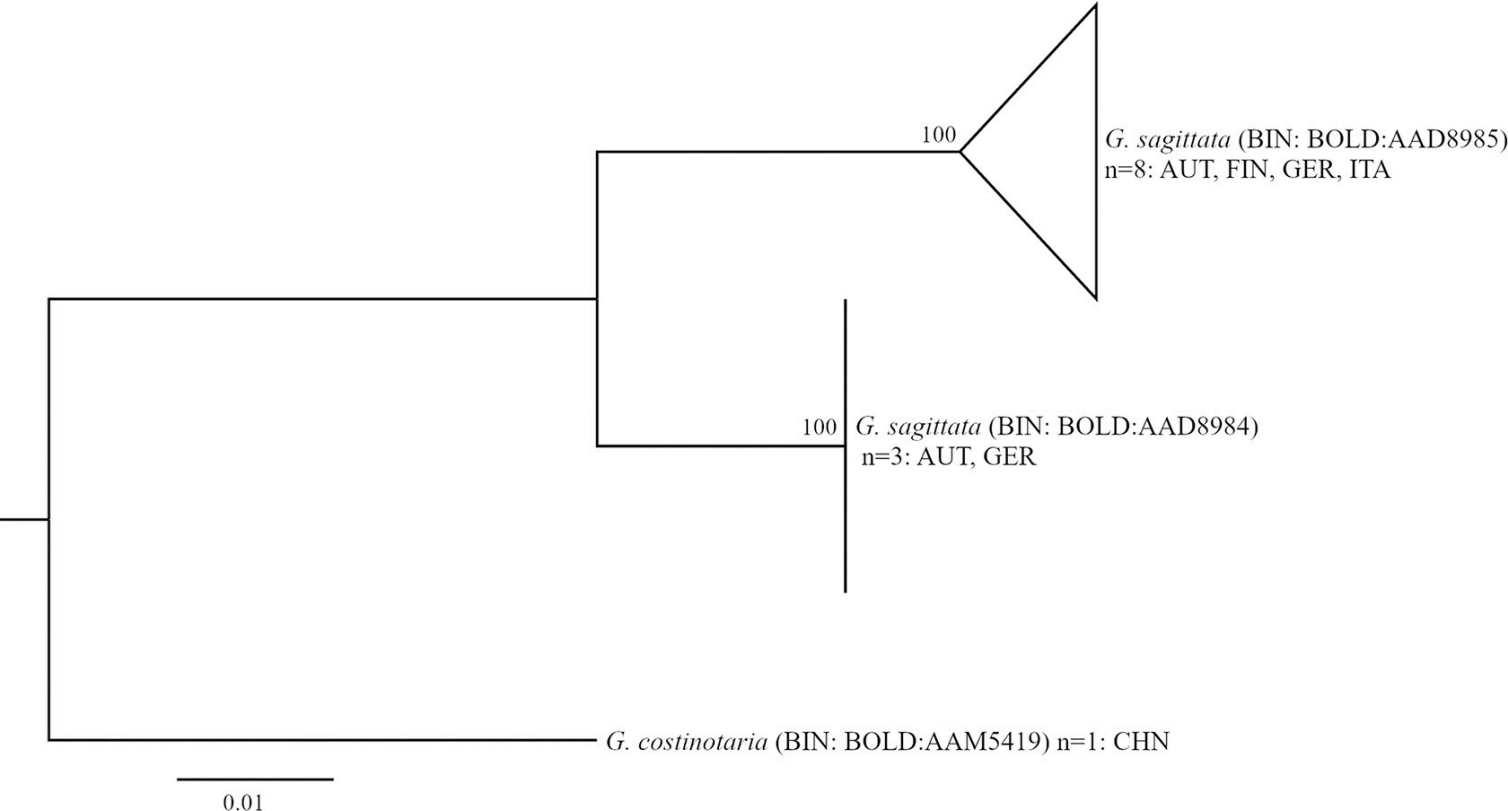
Phylogenetic relationships among BINs of *Gagitodes sagittata*, *G*. *costinotaria*. Neighbor Joining trees based on K2P distances (with pairwise deletion of missing/ambiguous characters), n = sample size; origin of samples indicated with ISO 3166–1 alpha-3 three-letter country code.

**Fig 8:**
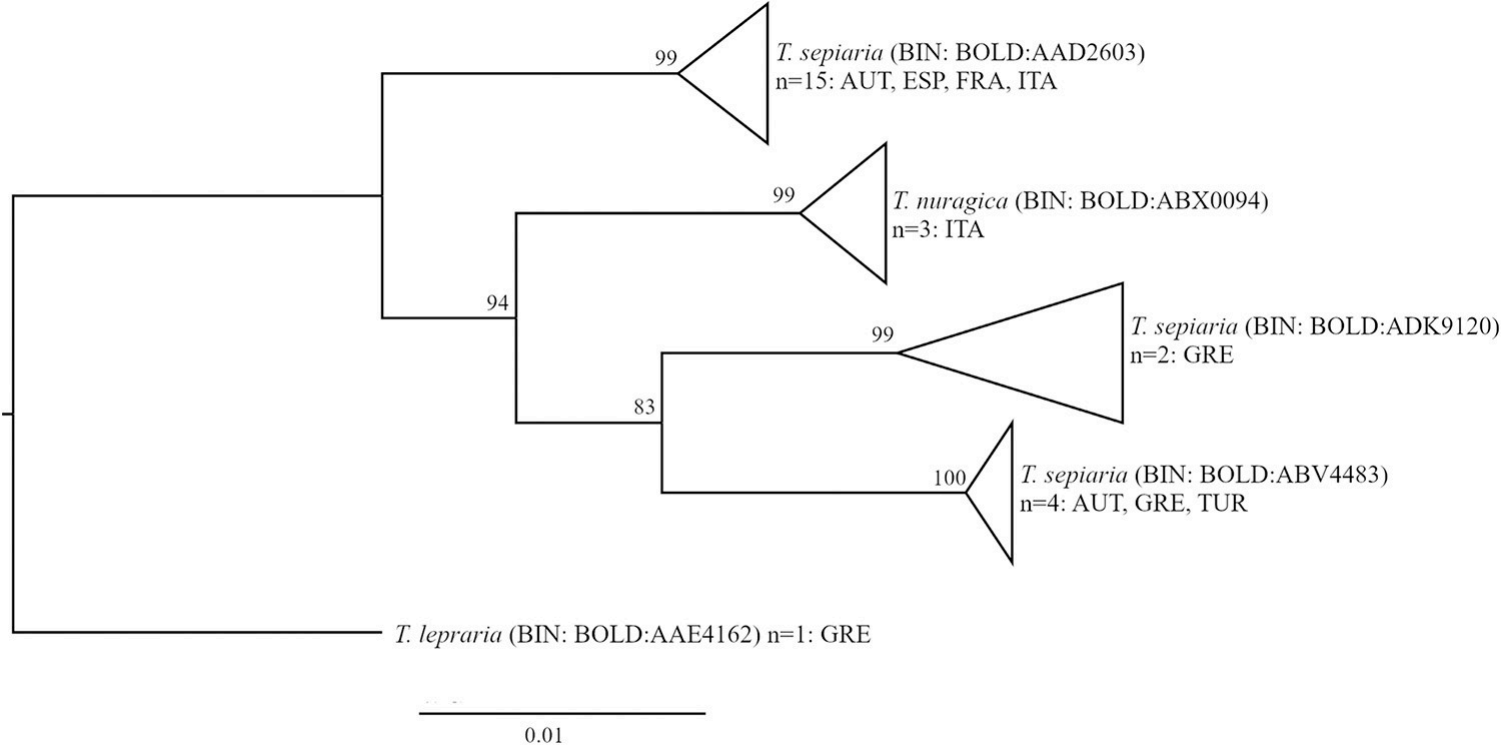
Phylogenetic relationships among BINs of *Tephronia sepiaria*, *T*. *nuragica* and *T*. *lepraria*. Neighbor Joining trees based on K2P distances (with pairwise deletion of missing/ambiguous characters), n = sample size; origin of samples indicated with ISO 3166–1 alpha-3 three-letter country code.

Based on the ASAP method, 396 morphospecies (83.1% of the Austrian geometrid species) were assigned to unique MOTUs (Fig 4). For a total of 61 morphospecies, ASAP did not discriminate between two or more morphospecies, and 22 morphospecies were split into two MOTUs (Fig 4). Three morphospecies experienced both MOTU splitting and MOTU sharing (*Rheumaptera subhastata*, *Sciadia tenebraria*, *Sciadia zelleraria*; S1 Table). Differences from BIN results concerned mainly the species-poor subfamilies (Geometrinae and Archiearinae), where ASAP achieved higher congruence with morphospecies delimitation than did the BIN method (Table 2). Threshold distances for MOTU-partitioning varied among subfamilies from K2P = 1.2% to K2P = 5.3% (Geometrinae, 5.3%; Archiearinae, 1.2%; Ennominae, 3.0%; Larentiinae, 4.1%; Sterrhinae, 2.2%).

**Fig 9:**
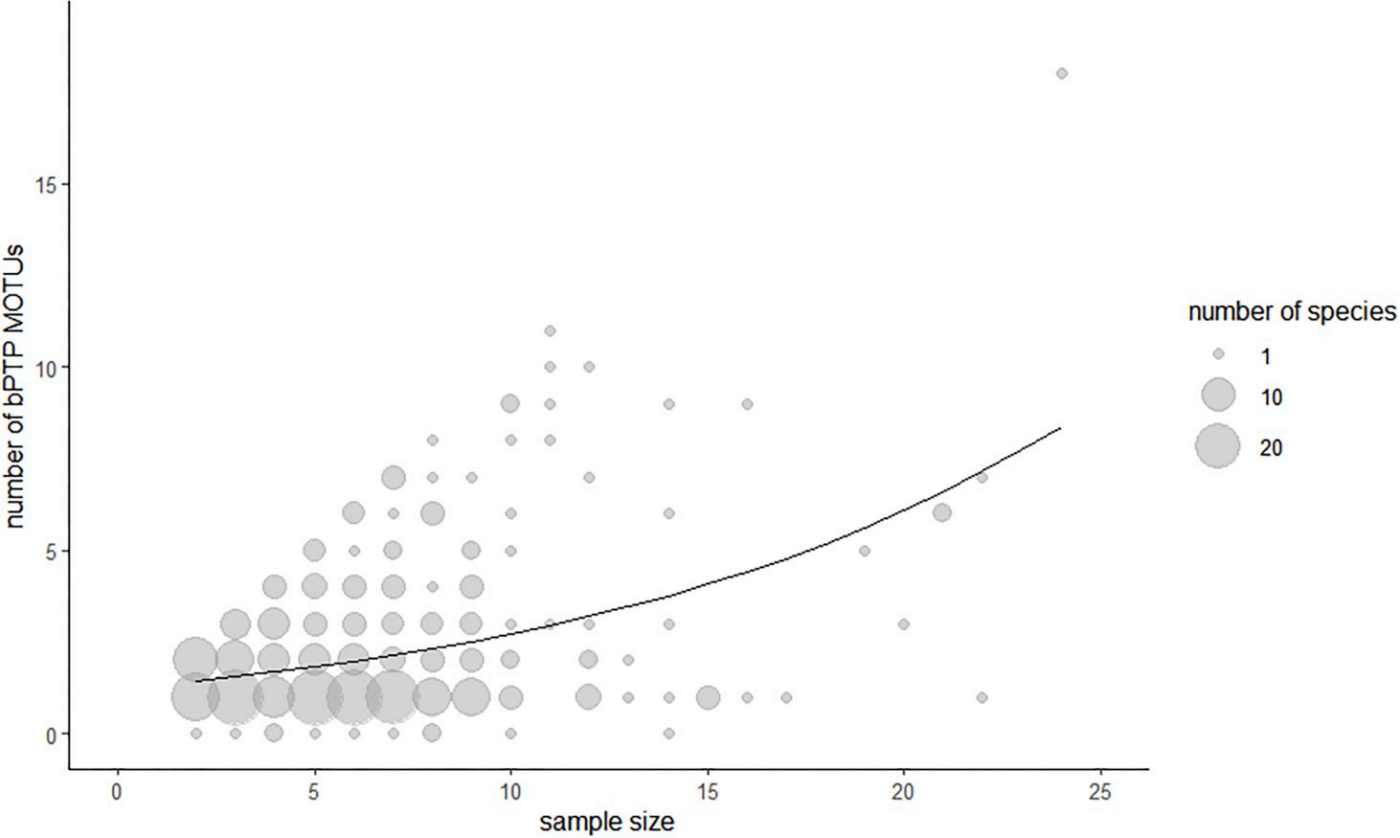
Increase of bPTP splitting with sample size per species. For species represented by more than one sample, the number of bPTP MOTUs is plotted against sample size. Dot size represents the number of species with identical values. The line illustrates the relationship predicted by the generalized linear model (predicted number of bPTP MOTUs = exp(0.233539 + sample size * 0.078961)).

The bPTP analysis resulted in the discrimination of 948 MOTUs. Only 282 morphospecies (59.2%) were assigned to individual unique MOTUs, while 181 morphospecies (36.6%) were split into multiple MOTUs and 20 morphospecies (4.2%) shared their MOTU with another species (Fig 4). Seven of these morphospecies showed both MOTU splitting and MOTU sharing (*Chlorissa cloraria*, *Chlorissa viridata*, *Elophos caelibaria*, *Rheumaptera subhastata*, *Sciadia tenebraria*, *Sciadia zelleraria*, *Thera cembrae*, *Thera obeliscata*; S1 Table). Five morphospecies were split into ten or more MOTUs (highest MOTU numbers:18 MOTUs in *Eupithecia virgaureata*, 12 MOTUs in *Charissa supinaria*, 11 MOTUs in *Eupithecia abietaria* and in *Operophtera brumata*, and10 MOTUs in *Eupithecia intricata*). Congruence between MOTUs and morphospecies was similarly poor across all subfamilies (Table 2). We found bPTP partitioning to be positively correlated with sampling effort, as the number of bPTP-MOTUs per species increased significantly with sample size per species (Fig 9; GLM: est. = 0.08, z = 8.78, p < 10^−16^, based on n = 409 species with sample size > 1).

## Discussion

### MOTU delimitation and morphospecies identification

The diversity of European Macrolepidoptera has been a longstanding research focus and is considered to be thoroughly recorded and characterized [71, 72]. This is also true for the family Geometridae, where the high quality and intensity of taxonomic work [31–36, 41, 42] results in morphospecies classifications that obviously also provide a good picture of the underlying genetic structure and diversity, although certain cases still remain disputable (e.g. species pairs *Chlorissa viridata/cloraria*, *Thera cembrae/obeliscata* and a few others). In our study, approximately 85% of morphospecies were represented by unique BINs and ASAP-MOTUs, while potential cases of cryptic diversity were suggested in 10% (BIN) or 5% (ASAP) of the Austrian species. Together with those of the BIN-sharing species whose sequences nonetheless cluster by species in phylogenetic trees (Table 1), this amounts to a total of 464 out of 476 species (97%) that can be identified by their COI sequence. These results correspond well with those of previous barcoding studies in Geometridae [37, 38], as well as with the success rates of genetic species identification that were achieved in Central European Ensifera (100%; [14]), European Apoidea (99%; [73]), Austrian Noctuoidea (98%; [20]), Northern European tachinid flies (93%; [74]), German Heteroptera (92%; [75]), European Coleoptera (92%; [76]), German Neuroptera (90%; [77]), European Odonata (88%; [13]) and European butterfly species (85% [78]). These successes in DNA-based species identification constitute a promising groundwork for monitoring studies that can for instance employ metabarcoding approaches to efficiently analyze insect diversity from Malaise trap or environmental DNA samples in a high throughput manner [79].

In the present study, BIN and ASAP performed similarly well in terms of congruence between reconstructed MOTUs and morphological taxonomy. Being embedded in the BOLD system, BIN makes use of the enormous amount of data in the database in MOTU construction and delimitation. While this is certainly an asset, the limited possibilities to curate the public data (e.g. by editing incorrect taxonomic notation) or to edit the alignment prior to analyses are undesirable constraints. In the framework of the current “Biodiversity Europe (BGE)” project, however, a comprehensive curation of the reference library on BOLD is aimed at a pan-European level. Our results suggest ASAP as a promising, fast alternative for MOTU delimitation and species identification that can be used with more flexibility in alignment construction. In cases of inconsistency with morphospecies classification, ASAP had a stronger tendency to morphospecies lumping than BIN, while BIN performed a higher rate of morphospecies splitting than ASAP (Fig 4). Similarly, BIN splits were far more common than BIN merges in a large dataset of Canadian spiders [56]. Between the two methods, their relative merits depend on the goal: While BIN seems to be more sensitive than ASAP to cryptic diversity but is perhaps prone to over-splitting, this does not impair species identification, where MOTU splitting is less problematic than MOTU sharing.

In contrast, the performance of bPTP as a species identification method was rather poor, as the number of MOTUs identified by bPTP was about twice as high as the number of morphospecies in the dataset, and was furthermore sensitive to sample size. Oversplitting by bPTP has also been observed in other datasets across various taxa [80–82].

### Incongruences between MOTUs and morphospecies: Sharing and splitting

Approximately 5% of the Austrian geometrid species cannot be identified based on the BIN system, as they share their BINs with one or two other, typically congeneric species (Table 1). One of the BIN sharing dyads was separated by ASAP. In many cases, the BIN sharing species also share DNA barcodes (i.e., have identical haplotypes), or sequences do not cluster by species in phylogenetic reconstructions (Table 1). However, with two exceptions, the BIN sharing species can be discriminated unambiguously based on morphological traits (see Table 1 for distinguishing traits) and likely represent young but distinct species. In some of these cases, present or past hybridization (genetic introgression) may be responsible for interspecific haplotype sharing (Table 1). In contrast, *Chlorissa cloraria* and *C*. *viridata* are difficult if not impossible to distinguish morphologically and share identical DNA barcodes, suggesting possible synonymy. Likewise, *Thera cembrae* and *T*. *obeliscata* are morphologically indistinguishable, although different in host plant use, and share identical DNA barcodes. *Thera cembrae* has been considered as possibly conspecific with *T*. *obeliscata* [36, 69] or as subspecies of *T*. *variata* [70]. The genetic data (Fig 5) refute the latter proposition, but are concordant with synonymy of the two species.

Approximately 10% of the morphospecies investigated in this study were split into multiple BINs, with intraspecific K2P distances exceeding 3% in 23 species (Table 3). Some of the detected BIN splits, especially those with large K2P distance, may be taxonomically relevant and need to be tested for support by nuclear genetic data and phenotypic traits. Most of the intraspecific BINs, which were detected in Austria, are shared with other European specimens of the same species (Table 3). In two cases, Austrian specimens share BINs with geographically more distant specimens: four Austrian samples of *Alcis repandata* are assigned to a BIN together with conspecific specimens from the Altai mountains [83], and one of the BINs assigned to Austrian *Eupithecia plumbeolata* is shared with the Asian species *E*. *nomogrammata*.

Furthermore, a new BIN was discovered in the species *Elophos operaria*. This BIN (BOLD:AEE3881) is composed of a single sample that was collected from the type locality of *E*. *operaria hoefneri* (Rebel, 1903). Müller et al. [32], however, found no morphological support for subspecies recognition.

A new BIN (BOLD:AEE3670) was also discovered in *Agriopis bajaria*, constituted by a sample from North-Eastern Austria. The remaining four specimens of this species were collected in Western and Eastern Austria and share their BIN with specimens from Southern Italy, western Mediterranean, Central-South-Eastern Europe and Lebanon.

**Fig 10:**
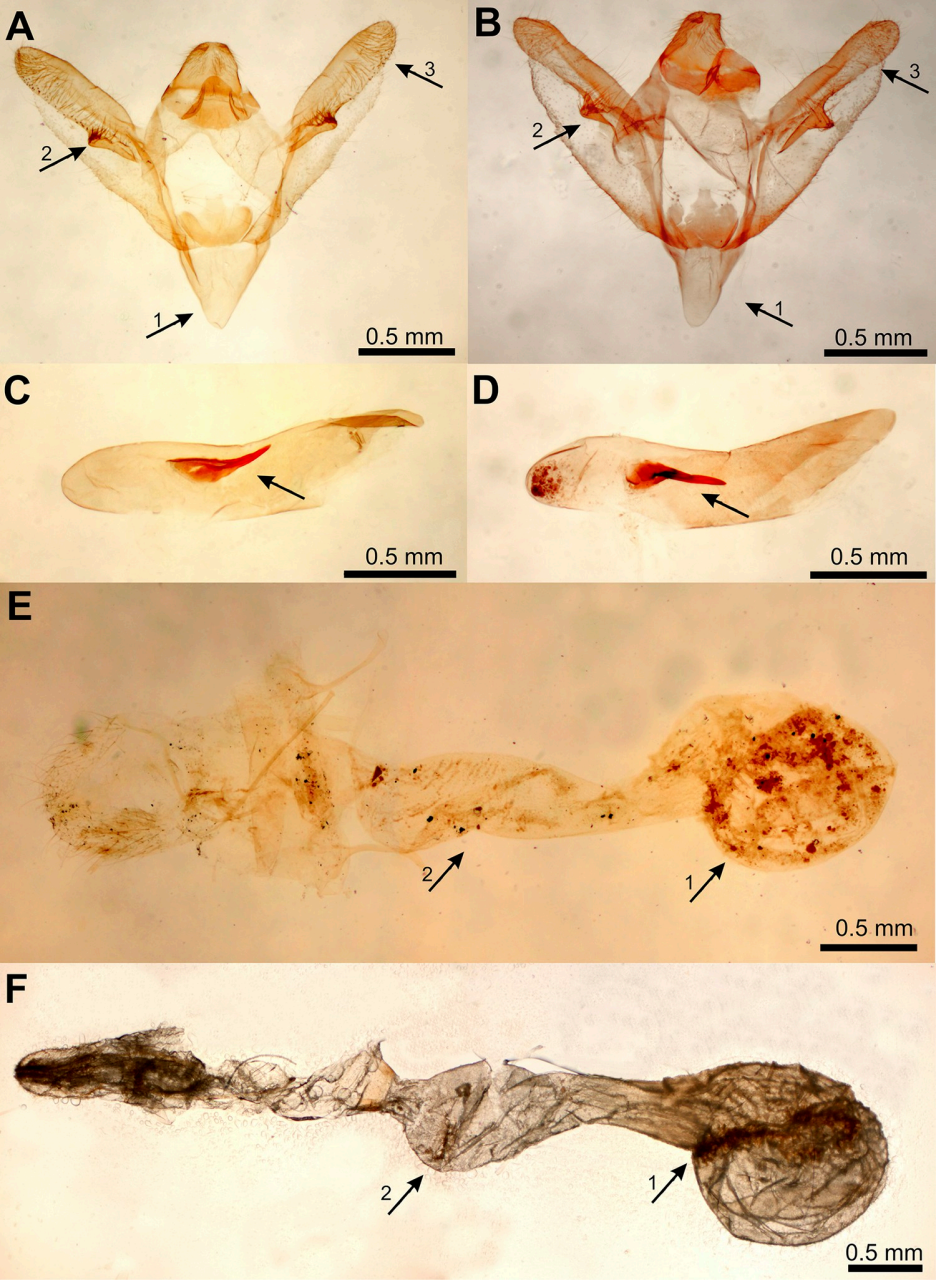
Genital preparations of *Tephronia sepiaria*. Genitalia of males in BIN: BOLD:AAD2603 (A) and BIN: BOLD:ABV4483 (B) differ in saccus width (arrow 1), shape of the valva (arrow 2) as well as the number of hairs on and the form of the distinct process on the central part of the valva (arrow 3). Aedeagi of males in BIN: BOLD:AAD2603 (C) and BIN: BOLD:ABV4483 (D) differ in the shape of the cornutus. Genitalia of females in BIN: BOLD:AAD2603 (E) and BIN: BOLD:ABV4483 (F) differ in the form of the signa (arrow 1) and the form of the ductus bursae (arrow 2). Photos by Andreas Eckelt. Specimens: (A, C) Italy, South Tyrol, Naturns, beneath Ladurner; 46.653, 10.975; 650 m; 06.07.2014; leg. P. Huemer. col. TLMF, G 1476 m; sample ID: TLMF Lep 14873. (B, D) Austria, Lower Austria, Bad Vöslau, Harzberg, Steinbruch; 47.9696, 16.1912; 395m; 25.7.2019; leg. C. Wieser; col. KLM, Gig. 12m; sample ID:KLM Lep 08783. (E) Italy, South Tyrol, Montiggl, Kleiner Priol; 46.428, 11.300; 643 m; 30.06.2010; leg. P. Huemer. col. TLMF, G 1478 f; sample ID: TLMF Lep 02391. (F) Austria, Lower Austria, Bad Vöslau, Harzberg, Steinbruch; 47.9696, 16.1912; 395m; 25.7.2019; leg. C. Wieser; col. KLM, GU 21/1529f.

In *Tephronia sepiaria*, specimens collected in Western Austria were assigned to a BIN shared by samples from Italy, France and Spain. In contrast, the *T*. *sepiaria* sample from Eastern Austria belongs to a BIN (BOLD:ABV4483) which has initially been defined based on specimens from central Turkey and for which subspecies status has been taken into consideration [32]. The BIN has then been detected in Greece (Hausmann, unpublished), and is now for the first time reported from Central Europe. A phylogenetic tree based on COI barcode sequences of *T*. *sepiaria* and its closest relatives *T*. *nuragica* and *T*. *lepraria* shows *T*. *sepiaria* separated in Eastern and Western clades and paraphyletic in relation to *T*. *nuragica*, which is a species endemic to Corsica and Sardinia (Fig 8). Examination of genitalia morphology in specimens of the two *T*. *sepiaria* BINs (n = 1 male and 1 female of each BIN) revealed minor differences (Fig 10). Further morphological data need to be collected in order to determine whether these morphological differences are BIN-specific, and the taxonomy of *T*. *sepiaria* and *T*. *nuragica* should be further investigated with both genetic and morphological data.

Another interesting taxonomic problem exists in the genus *Crocallis*. The species *C*. *elinguaria* was found to consist of four BINs (BOLD:AAB0677, BOLD:ACE7622, BOLD:ACF1889, BOLD:AAE4231; [31]), of which BOLD:AAB0677 is widespread across Europe, while BOLD:ACE7622 and BOLD:ACF1889 are found in southern Italy. Twelve Austrian specimens of *C*. *elinguaria* all assign to BIN BOLD:AAB0677. The last of the four BINs, BOLD:AAE4231, contains specimens from South-Eastern Europe that have been identified as *C*. *elinguaria*, but the same BIN is shared with a Turkish species, *C*. *inexpectata*. The taxonomy of the group is currently uncertain, and specimens in this BIN are often referred to as *Crocallis* sp. [31]. Six of the Austrian samples were assigned to this BIN, extending the known distribution of BOLD:AAE4231 to Lower Austria and Carinthia.

**Fig 11:**
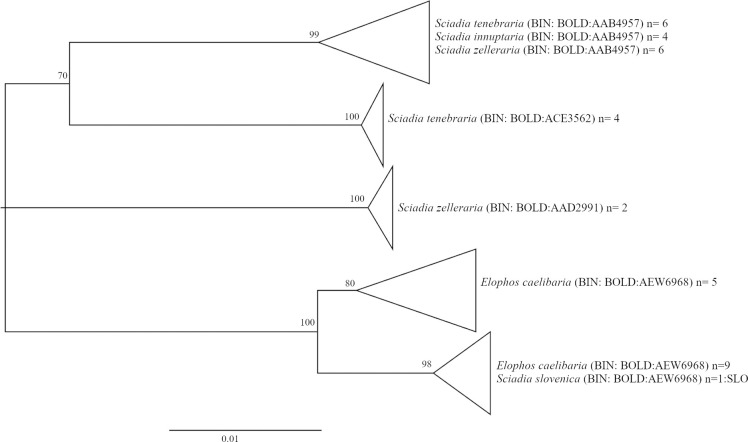
Phylogenetic relationships among BINs and species of *Sciadia* spp. and *Elophos caelibaria*. Neighbor Joining trees based on K2P distances (with pairwise deletion of missing/ambiguous characters), n = sample size; samples are of Austrian origin, except for *Sciadia slovenica* (from Slovenia).

The majority of the intraspecific BINs seem to overlap geographically, as their sampling locations do not encompass mutually exclusive areas (Table 3). The classification of ‘overlapping’ versus ‘non-overlapping’ distributions in Table 3 are based on current BOLD entries, and may prove incorrect when larger sample sizes increase the spatial resolution. For instance, only three DNA barcodes of *Elophos operaria* are available to date, and since the distribution of one BIN (2 samples) does not encompass the location of the other BIN, we scored the two BINs as ‘non-overlapping’. Overlapping MOTUs may be reproductively isolated from each other and represent cryptic species; alternatively, divergent mitochondrial lineages may evolve in consequence of Wolbachia infection [84], represent admixture of historically isolated lineages [85] or introgression from other species [86], but can also evolve in large panmictic populations in the absence of population structure [87]. The coincidence of BIN-sharing and BIN-splitting detected in some species of the present study suggests introgression as the source of intraspecific mitochondrial divergence. *Sciadia tenebraria* and *S*. *zelleraria* are both split into multiple intraspecific BINs, some of which are shared reciprocally and with *S*. *innuptaria* (Fig 11). Since hybridization of species in the genus *Sciadia* is common [32], introgression of haplotypes is a likely explanation for this BIN pattern. Similarly, Austrian *Rheumaptera subhastata* are split into two BINs, one of which is more closely related to the sister species *R*. *hastata* than to the conspecific BIN, again a possible consequence of introgression [36]. However, detailed integrative analyses are necessary in each case to elucidate the underlying causes and potential taxonomic implications of the observed BIN splits.

## Conclusions

DNA barcoding and sequence analysis of the complete set of Austrian Geometridae species revealed a high potential for accurate DNA-based species identification, which is promising groundwork for applications with bulk samples or environmental DNA. The present study also identified 8 species dyads and triads, within which species cannot be distinguished based on COI barcode region sequences (Table 1). Some of them involve morphologically distinct species known to be prone to hybridization. The high level of congruency between morphospecies taxonomy and genetic identification in the present study is, at least in part, due to a history of thorough taxonomic work on the European fauna. We expect that more incongruencies will be detected when studies are extended to less well studied areas [88]. Finally, the table of species investigated in the current study (S1 Table) represents an updated checklist of the geometrid moths of Austria.

## Supporting information

S1 Table List of species included in this study, including the number and origin of samples used in the analyses, MOTU delimitation results as well as the minimum distance to, and identity of, the nearest neighbor species. For each species delimitation method, the resulting MOTUs were given consecutive numbers (IDs of BINs, ASAP-MOTUs and bPTP-MOTUs). MOTUs shared among species are highlighted in yellow.
(XLSX)

S2 Table Results of MOTU delimitation analyses, broken down by subfamilies. (DOCX)

S1 Text Genbank accession numbers. (DOCX)

## References

[1] Cervellini M, Zannini P, Di Musciano M, Fattorini S, Jiménez-Alfaro B, Rocchini D, et al. A grid-based map for the Biogeographical Regions of Europe. Biodivers Data J. 30AD;8: e53720. doi: 10.3897/BDJ.8.e53720 32684779 PMC7340631

[2] Sauberer N, Prinz M, Essl F. Österreichs Klima, Geographie und Landbedeckung im Überblick. Denisia. 2017;39: 27–34.

[3] Rabitsch W, Graf W, Huemer P, Kahlen M, Komposch C, Paill W, et al. Biogeography and ecology of endemic invertebrate species in Austria: A cross-taxon analysis. Basic Appl Ecol. 2016;17: 95–105. doi: 10.1016/J.BAAE.2015.11.002

[4] Huemer P. Die Schmetterlinge Österreichs (Lepidoptera). Innsbruck: Tiroler Landesmuseen; 2013.

[5] Karsholt O, Razowski J. The Lepidoptera of Europe: a distributional checklist. Brill Academic Pub; 1996.

[6] Geiser E. How Many Animal Species are there in Austria? Update after 20 Years. Acta Zoobot Austria. 2018;155: 1–18.

[7] Zangl L, Schäffer S, Daill D, Friedrich T, Gessl W, Mladinić M, et al. A comprehensive DNA barcode inventory of Austria’s fish species. PLoS One. 2022;17: e0268694. doi: 10.1371/journal.pone.0268694 35679240 PMC9182252

[8] Zangl L, Daill D, Schweiger S, Gassner G, Koblmüller S. A reference DNA barcode library for Austrian amphibians and reptiles. PLoS One. 2020;15: e0229353. doi: 10.1371/journal.pone.0229353 32163447 PMC7067431

[9] Lewisch E, Solymos V, Waldner K, L van der V, Harl J, Bakran-Lebl K, et al. Acanthocephalan parasites collected from Austrian fishes: molecular barcoding and pathological observations. Dis Aquat Organ. 2020;139: 103–111. https://www.int-res.com/abstracts/dao/v139/p103-111/ doi: 10.3354/dao03471 32351241

[10] Reier S, Sattmann H, Schwaha T, Fuehrer H-P, Haring E. Unravelling the hidden biodiversity–the establishment of DNA barcodes of fish-parasitizing Acanthocephala Koehlreuther, 1771 in view of taxonomic misidentifications, intraspecific variability and possible cryptic species. Parasitology. 2020;147: 1499–1508. doi: 10.1017/S0031182020001316 32741413 PMC7677897

[11] Resch MC, Shrubovych J, Bartel D, Szucsich NU, Timelthaler G, Bu Y, et al. Where Taxonomy Based on Subtle Morphological Differences Is Perfectly Mirrored by Huge Genetic Distances: DNA Barcoding in Protura (Hexapoda). PLoS One. 2014;9: e90653. doi: 10.1371/journal.pone.0090653 24609003 PMC3946556

[12] Shrubovych J, Bartel D, Szucsich NU, Resch MC, Pass G. Morphological and Genetic Analysis of the *Acerentomon doderoi* Group (Protura: Acerentomidae) with Description of *A*. *christiani* sp. nov. PLoS One. 2016;11: e0148033. doi: 10.1371/journal.pone.0148033 27073924 PMC4830551

[13] Geiger M, Koblmüller S, Assandri G, Chovanec A, Ekrem T, Fischer I, et al. Coverage and quality of DNA barcode references for Central and Northern European Odonata. PeerJ. 2021;9: e11192. doi: 10.7717/peerj.11192 33986985 PMC8101477

[14] Hawlitschek O, Morinière J, Lehmann GUC, Lehmann AW, Kropf M, Dunz A, et al. DNA barcoding of crickets, katydids and grasshoppers (Orthoptera) from Central Europe with focus on Austria, Germany and Switzerland. Mol Ecol Resour. 2017;17: 1037–1053. doi: 10.1111/1755-0998.12638 27863033

[15] Zangl L, Glatzhofer E, Schmid R, Randolf S, Koblmüller S. DNA barcoding of Austrian snow scorpionflies (Mecoptera, Boreidae) reveals potential cryptic diversity in *Boreus westwoodi*. PeerJ. 2021;9: e11424. 34040896 10.7717/peerj.11424 PMC8127955

[16] Zittra C, Wöss G, Van der Vloet L, Bakran-Lebl K, Shahi Barogh B, Sehnal P, et al. Barcoding of the Genus *Culicoides* (Diptera: Ceratopogonidae) in Austria—An Update of the Species Inventory Including the First Records of Three Species in Austria. Pathogens. 2020;9: 406. doi: 10.3390/pathogens9050406 32456256 PMC7280969

[17] Zittra C, Moog O, Christian E, Fuehrer H-P. DNA-aided identification of *Culex* mosquitoes (Diptera: Culicidae) reveals unexpected diversity in underground cavities in Austria. Parasitol Res. 2019;118: 1385–1391. doi: 10.1007/s00436-019-06277-y 30919062 PMC6478630

[18] Bakran-Lebl K, Pree S, Brenner T, Daroglou E, Eigner B, Griesbacher A, et al. First Nationwide Monitoring Program for the Detection of Potentially Invasive Mosquito Species in Austria. Insects. 2022;13. doi: 10.3390/insects13030276 35323574 PMC8949374

[19] Huemer P, Wiesmair Benjamin. DNA-Barcoding der Tagfalter (Lepidoptera, Papilionoidea) Österreichs—unbekannte genetische Vielfalt im Zentrum Europas. Wiss Jahrb Tirol Landesmuseen. 2017;10: 8–33.

[20] Huemer P, Wieser C, Stark W, Hebert PDN, Wiesmair B. DNA barcode library of megadiverse Austrian Noctuoidea (Lepidoptera)–a nearly perfect match of Linnean taxonomy. Biodivers Data J. 2019;7. 10.3897/BDJ.7.e37734 PMC6694074 31423084

[21] Huemer P, Karsholt O, Aarvik L, Berggren K, Bidzilya O, Junnilainen J, et al. DNA barcode library for European Gelechiidae (Lepidoptera) suggests greatly underestimated species diversity. Zookeys. 2020;921: 141–157. doi: 10.3897/zookeys.921.49199 32256152 PMC7109146

[22] Lopez-Vaamonde C, Kirichenko N, Cama A, Doorenweerd C, Godfray HCJ, Guiguet A, et al. Evaluating DNA Barcoding for Species Identification and Discovery in European Gracillariid Moths. Front Ecol Evol. 2021;9. doi: 10.3389/fevo.2021.626752

[23] Huemer P, Mutanen M, Sefc KM, Hebert PDN. Testing DNA Barcode Performance in 1000 Species of European Lepidoptera: Large Geographic Distances Have Small Genetic Impacts. PLoS One. 2014;9: e115774. doi: 10.1371/journal.pone.0115774 25541991 PMC4277373

[24] Huemer P, Hebert PDN. DNA Barcode Bibliothek der Schmetterlinge Südtirols und Tirols (Italien, Österreich)–Impetus für integrative Artdifferenzierung im 21. Jahrhundert. Gredleriana (Bolzano). 2016;16: 141–146.

[25] Scoble M, Hausmann A. Online list of valid and available names of the Geometridae of the World. Lepidoptera Barcode of Life. 2007. https://geometroidea.smns-bw.org/archive/48

[26] Rajaei H, Hausmann A, Scoble M, Wanke D, Plotkin D, Brehm G, et al. An online taxonomic facility of Geometridae (Lepidoptera), with an overview of global species richness and systematics. Integr Syst. 2022;5. doi: 10.18476/2022.577933

[27] Scoble M. Geometrid Moths of the World. CSIRO Publishing; 1999. doi: 10.1071/9780643101050

[28] Sihvonen P, Mutanen M, Kaila L, Brehm G, Hausmann A, Staude HS. Comprehensive Molecular Sampling Yields a Robust Phylogeny for Geometrid Moths (Lepidoptera: Geometridae). PLoS One. 2011;6: e20356. doi: 10.1371/journal.pone.0020356 21673814 PMC3106010

[29] Murillo-Ramos L, Brehm G, Sihvonen P, Hausmann A, Holm S, Reza Ghanavi H, et al. A comprehensive molecular phylogeny of Geometridae (Lepidoptera) with a focus on enigmatic small subfamilies. PeerJ. 2019;7: e7386. doi: 10.7717/peerj.7386 31523494 PMC6716565

[30] Hausmann A, Sihvonen P. Revised, annotated systematic checklist of the Geometridae of Europe and adjacent areas, Vols 1–6. In: Hausmann A, Sihvonen P, editors. Ennominae II. Brill; 2019. pp. 795–871. doi: 10.1163/9789004387485_016

[31] Sihvonen P, Skou P. Ennominae I. Brill; 2015. doi: 10.1163/9789004265738

[32] Müller B, Erlacher S, Hausmann A, Rajaei H, Sihvonen P, Skou P. Ennominae II. Brill; 2019. doi: 10.1163/9789004387485

[33] Mironov V. Larentiinae II. Brill; 2003. doi: 10.1163/9789004308633

[34] Hausmann A. Sterrhinae. Brill; 2004. doi: 10.1163/9789004322554

[35] Hausmann A. Introduction to the series. Archiearinae, Oenochrominae, Geometrinae. Brill; 2001. doi: 10.1163/9789004322547

[36] Hausmann A, Viidalepp J. Larentiinae I. Brill; 2012. doi: 10.1163/9789004260979

[37] Hausmann A, Godfray HCJ, Huemer P, Mutanen M, Rougerie R, van Nieukerken EJ, et al. Genetic Patterns in European Geometrid Moths Revealed by the Barcode Index Number (BIN) System. PLoS One. 2013;8: e84518. doi: 10.1371/journal.pone.0084518 24358363 PMC3866169

[38] Hausmann A, Haszprunar G, Hebert PDN. DNA Barcoding the Geometrid Fauna of Bavaria (Lepidoptera): Successes, Surprises, and Questions. PLoS One. 2011;6: e17134. doi: 10.1371/journal.pone.0017134 21423340 PMC3040642

[39] Skou P, Stüning D, Sihvonen P. Revision of the West-Mediterranean geometrid genus *Ekboarmia*, with description of a new species from Portugal (Lepidoptera, Geometridae, Ennominae). Nota Lepidopterol. 2017;40: 39–63. doi: 10.3897/nl.40.10440

[40] Müller B. *Biston* rosenbaueri sp. n. (Lepidoptera, Geometridae, Ennominae) from the Balkan Peninsula. Nota Lepidopterol. 2018;41: 207–213. doi: 10.3897/nl.41.25099

[41] Hausmann A. Revision of the West Palaearctic *Idaea nocturna* species group (Lepidoptera, Geometridae, Sterrhinae). Mitteilungen der Münchner Entomologischen Gesellschaft. 2020;110: 71–80.

[42] Guerrero J-J, Hausmann A, Ortiz AS. Description of *Idaea josephinae* sp. n. from the Iberian Peninsula (Lepidoptera: Geometridae). Zootaxa. 2021;4990. doi: 10.11646/zootaxa.4990.2.10 34186754

[43] Scalercio S, Infusino M, Huemer P, Mutanen M. Pruning the Barcode Index Numbers tree: Morphological and genetic evidence clarifies species boundaries in the *Eupithecia conterminata* complex (Lepidoptera: Geometridae) in Europe. Journal of Zoological Systematics and Evolutionary Research. 2021;59: 1962–1981. doi: 10.1111/jzs.12568

[44] Huemer P, Friebe GJ, Wiesmair B, Mayr T, Hiermann U, Siegel C. Zur Verbreitung von *Perizoma juracolaria* (Lepidoptera, Geometridae, Larentiinae)–Erstnach- weise aus Österreich, Liechtenstein und Italien. inatura–Forschung online. 2015;25: 1–9.

[45] Stark W. Neunachweise von Lepidoptera (Schmetterlinge) für Mitteleuropa, Österreich und Niederösterreich sowie Bestätigungen von seltenen und fraglichen Arten–Ergebnisse der Initiative„Leuchtturmprojekt Schmetterlinge. Naturkundliche Mitteilungen aus den Landessammlungen Niederösterreich. 2022;32: 5–20.

[46] Dirnböck T, Essl F, Rabitsch W. Disproportional risk for habitat loss of high-altitude endemic species under climate change. Glob Chang Biol. 2011;17: 990–996. doi: 10.1111/j.1365-2486.2010.02266.x

[47] Huemer P, Rüdisser J, Hiermann U, Lechner K, Mayr T, Ortner A, et al. Rote Liste gefährdeter Schmetterlinge Vorarlbergs (Neubearbeitung). Rote Listen Vorarlbergs. 2022;11: 1–210.

[48] Embacher G. Rote Liste der Großschmetterlinge Salzburgs. 2., neu bearbeitete Auflage. Naturschutzbeiträge. 1991;7: 5–61.

[49] Hauser E. Rote Liste der Groß-Schmetterlinge Oberösterreichs (Stand 1995). Beitr Naturk Oberösterreichs. 1996;4: 53–66.

[50] Wieser C. Schmetterlinge (Insecta: Lepidoptera). In: Komposch C, editor. Rote Listen gefährdeter Tiere Kärntens. Klagenfurt: Naturwissenschaftlicher Verein für Kärnten; 2023.

[51] Comtet T, Sandionigi A, Viard F, Casiraghi M. DNA (meta)barcoding of biological invasions: a powerful tool to elucidate invasion processes and help managing aliens. Biol Invasions. 2015;17: 905–922. doi: 10.1007/s10530-015-0854-y

[52] Ratnasingham S, Hebert PDN. BOLD: The Barcode of Life Data System (http://www.barcodinglife.org). Mol Ecol Notes. 2007;7: 355–364. doi: 10.1111/j.1471-8286.2007.01678.x 18784790 PMC1890991

[53] Robinson GS. Preparation of slides of Lepidoptera genitalia with special reference to the Microlepidoptera. Entomol Gaz. 1976;27: 127–132.

[54] deWaard JR, Ivanova N V., Hajibabaei M, Hebert PDN. Assembling DNA Barcodes. 2008. pp. 275–294. doi: 10.1007/978-1-59745-548-0_15 18642605

[55] Hebert PDN, Penton EH, Burns JM, Janzen DH, Hallwachs W. Ten species in one: DNA barcoding reveals cryptic species in the neotropical skipper butterfly *Astraptes fulgerator*. Proceedings of the National Academy of Sciences. 2004;101: 14812–14817. doi: 10.1073/pnas.0406166101 15465915 PMC522015

[56] Blagoev GA, deWaard JR, Ratnasingham S, deWaard SL, Lu L, Robertson J, et al. Untangling taxonomy: a DNA barcode reference library for Canadian spiders. Mol Ecol Resour. 2016;16: 325–341. doi: 10.1111/1755-0998.12444 26175299

[57] Puillandre N, Brouillet S, Achaz G. ASAP: assemble species by automatic partitioning. Mol Ecol Resour. 2021;21: 609–620. doi: 10.1111/1755-0998.13281 33058550

[58] Zhang J, Kapli P, Pavlidis P, Stamatakis A. A general species delimitation method with applications to phylogenetic placements. Bioinformatics. 2013;29: 2869–2876. doi: 10.1093/bioinformatics/btt499 23990417 PMC3810850

[59] Zhang D, Gao F, Jakovlić I, Zou H, Zhang J, Li WX, et al. PhyloSuite: An integrated and scalable desktop platform for streamlined molecular sequence data management and evolutionary phylogenetics studies. Mol Ecol Resour. 2020;20: 348–355. doi: 10.1111/1755-0998.13096 31599058

[60] Katoh K, Standley DM. MAFFT Multiple Sequence Alignment Software Version 7: Improvements in Performance and Usability. Mol Biol Evol. 2013;30: 772–780. doi: 10.1093/molbev/mst010 23329690 PMC3603318

[61] Kalyaanamoorthy S, Minh BQ, Wong TKF, von Haeseler A, Jermiin LS. ModelFinder: fast model selection for accurate phylogenetic estimates. Nat Methods. 2017;14: 587–589. doi: 10.1038/nmeth.4285 28481363 PMC5453245

[62] Nguyen L-T, Schmidt HA, von Haeseler A, Minh BQ. IQ-TREE: A Fast and Effective Stochastic Algorithm for Estimating Maximum-Likelihood Phylogenies. Mol Biol Evol. 2015;32: 268–274. doi: 10.1093/molbev/msu300 25371430 PMC4271533

[63] Brooks ME, Kristensen K, Benthem KJ, van, Magnusson A, Berg CW, Nielsen A, et al. glmmTMB Balances Speed and Flexibility Among Packages for Zero-inflated Generalized Linear Mixed Modeling. R J. 2017;9: 378. doi: 10.32614/RJ-2017-066

[64] Kumar S, Stecher G, Li M, Knyaz C, Tamura K. MEGA X: Molecular Evolutionary Genetics Analysis across Computing Platforms. Mol Biol Evol. 2018;35: 1547–1549. doi: 10.1093/molbev/msy096 29722887 PMC5967553

[65] Rambaut A. FigTree. Rambaut, A., 2015. FigTree. Edinburgh, Scotland.: Rambaut, Andrew; 2015.

[66] The GIMP Development Team. GIMP. 2019.

[67] Harrison JWH. New hybrid Bistoninae. Entomologist. 1910;43: 197–198.

[68] Thomann H. *Codonia* hybr. *Kessleri* m. hybr. nov. (Lep., Geom.) = *C*. *pendularia* Cl. ♂ x *C*. *pupillaria* Hb. ♀. Mitt ent Ges Basel. 1955;5: 33–35.

[69] Haslberger A, Segerer AH. Systematische, revidierte und kommentierte Checkliste der Schmetterlinge Bayerns (Insecta: Lepidoptera). Mitteilungen der Münchner Entomologischen Gesellschaft. 2016;106: 1–336.

[70] Leraut P. Moths of Europe-Volume 2: Geometrid moths. Moths of Europe-Volume 2: Geometrid moths. Verrières-le-Buisson: Nature Art Planete; 2009.

[71] Hebert PDN, Cywinska A, Ball SL, deWaard JR. Biological identifications through DNA barcodes. Proc R Soc Lond B Biol Sci. 2003;270: 313–321. doi: 10.1098/rspb.2002.2218 12614582 PMC1691236

[72] Fibiger M, Hacker HH. Systematic List of the Noctuoidea of Europe (Notodontidae, Nolidae, Arctiidae, Lymantriidae, Erebidae, Micronoctuidae, and Noctuidae). Esperiana. 2005;11: 93–205.

[73] Wiemers M, Balletto E, Dincă V, Fric ZF, Lamas G, Lukhtanov V, et al. An updated checklist of the European Butterflies (Lepidoptera, Papilionoidea). Zookeys. 2018;811: 9–45. doi: 10.3897/zookeys.811.28712 30627036 PMC6323101

[74] Schmid‐Egger C, Straka J, Ljubomirov T, Blagoev GA, Morinière J, Schmidt S. DNA barcodes identify 99 per cent of apoid wasp species (Hymenoptera: Ampulicidae, Crabronidae, Sphecidae) from the Western Palearctic. Mol Ecol Resour. 2019;19: 476–484. doi: 10.1111/1755-0998.12963 30431229

[75] Pohjoismäki JLO, Kahanpää J, Mutanen M. DNA Barcodes for the Northern European Tachinid Flies (Diptera: Tachinidae). PLoS One. 2016;11: e0164933. doi: 10.1371/journal.pone.0164933 27814365 PMC5096672

[76] Raupach MJ, Hendrich L, Küchler SM, Deister F, Morinière J, Gossner MM. Building-Up of a DNA Barcode Library for True Bugs (Insecta: Hemiptera: Heteroptera) of Germany Reveals Taxonomic Uncertainties and Surprises. PLoS One. 2014;9: e106940. doi: 10.1371/journal.pone.0106940 25203616 PMC4159288

[77] Hendrich L, Morinière J, Haszprunar G, Hebert PDN, Hausmann A, Köhler F, et al. A comprehensive DNA barcode database for Central European beetles with a focus on Germany: adding more than 3500 identified species to BOLD. Mol Ecol Resour. 2015;15: 795–818. doi: 10.1111/1755-0998.12354 25469559

[78] Morinière J, Hendrich L, Hausmann A, Hebert P, Haszprunar G, Gruppe A. Barcoding Fauna Bavarica: 78% of the Neuropterida Fauna Barcoded! PLoS One. 2014;9: e109719. doi: 10.1371/journal.pone.0109719 25286434 PMC4186837

[79] Dincă V, Dapporto L, Somervuo P, Vodă R, Cuvelier S, Gascoigne-Pees M, et al. High resolution DNA barcode library for European butterflies reveals continental patterns of mitochondrial genetic diversity. Commun Biol. 2021;4: 315. doi: 10.1038/s42003-021-01834-7 33750912 PMC7943782

[80] Kirse A, Bourlat SJ, Langen K, Fonseca VG. Metabarcoding Malaise traps and soil eDNA reveals seasonal and local arthropod diversity shifts. Sci Rep. 2021;11: 10498. doi: 10.1038/s41598-021-89950-6 34006991 PMC8131643

[81] Blair C, Bryson RW. Cryptic diversity and discordance in single-locus species delimitation methods within horned lizards (Phrynosomatidae: *Phrynosoma*). Mol Ecol Resour. 2017;17: 1168–1182. doi: 10.1111/1755-0998.12658 28161911

[82] Hofmann EP, Nicholson KE, Luque-Montes IR, Köhler G, Cerrato-Mendoza CA, Medina-Flores M, et al. Cryptic Diversity, but to What Extent? Discordance Between Single-Locus Species Delimitation Methods Within Mainland Anoles (Squamata: Dactyloidae) of Northern Central America. Front Genet. 2019;10. doi: 10.3389/fgene.2019.00011 30804976 PMC6378269

[83] Wattier R, Mamos T, Copilaş-Ciocianu D, Jelić M, Ollivier A, Chaumot A, et al. Continental-scale patterns of hyper-cryptic diversity within the freshwater model taxon *Gammarus fossarum* (Crustacea, Amphipoda). Sci Rep. 2020;10: 16536. doi: 10.1038/s41598-020-73739-0 33024224 PMC7538970

[84] Huemer P, Hebert PDN, Mutanen M, Wieser C, Wiesmair B, Hausmann A, et al. Large geographic distance versus small DNA barcode divergence: Insights from a comparison of European to South Siberian Lepidoptera. PLoS One. 2018;13: e0206668. doi: 10.1371/journal.pone.0206668 30388147 PMC6214556

[85] Xiao J-H, Wang N-X, Murphy RW, Cook J, Jia L-Y, Huang D-W. Wolbachia infection and dramatic intraspecific mitochondrial DNA divergence in a fig wasp. Evolution (N Y). 2012;66: 1907–1916. doi: 10.1111/j.1558-5646.2011.01561.x 22671555

[86] Searle JB. Phylogeography—The History and Formation of Species. Avise John C. Harvard University Press, Cambridge, MA. 2000. Pp. 447 ISBN 0 674 66638 0. Heredity. 2000. doi: 10.1046/j.1365-2540.2000.0765d.x

[87] Koblmüller S, Nevado B, Makasa L, Van Steenberge M, Vanhove MPM, Verheyen E, et al. Phylogeny and phylogeography of *Altolamprologus*: ancient introgression and recent divergence in a rock-dwelling Lake Tanganyika cichlid genus. Hydrobiologia. 2017;791: 35–50. doi: 10.1007/s10750-016-2896-2

[88] Benham PM, Cheviron ZA. Divergent mitochondrial lineages arose within a large, panmictic population of the Savannah sparrow (*Passerculus sandwichensis*). Mol Ecol. 2019;28: 1765–1783. doi: 10.1111/mec.15049 30770598

