# Supplementary material for: A DNA barcode library of Austrian geometridae (Lepidoptera) reveals high potential for DNA-based species identification
Source: PLoS One. 2024 Mar 11;19(3):e0298025. doi: 10.1371/journal.pone.0298025 (PMC10927147; doi:10.1371/journal.pone.0298025)
Supplement: S2 Table — (DOCX) [file pone.0298025.s002.docx]

|  | Geometrinae | Archiearinae | Ennominae | Larentiinae | Sterrhinae |
| --- | --- | --- | --- | --- | --- |
| **Morphospecies** | 13 | 3 | 146 | 248 | 69 |
| **BINs** | 13 | 3 | 159 | 266 | 69 |
| **ASAP** |  |  |  |  |  |
| Number of MOTUs in most supported partition | 11 | 3 | 146 | 238 | 67 |
| ASAP score | 1.50 | 1.00 | 8.00 | 11.00 | 6.50 |
| Minimum and maximum number of MOTUs | 11 – 20 | 2 – 5 | 144 – 160 | 232 – 272 | 60 – 80 |
| **bPTP** |  |  |  |  |  |
| Number of MOTUs in most supported partition | 31 | 2 | 269 | 533 | 113 |
| Acceptance rate | 0.564 | 0.334 | 0.486 | 0.523 | 0.538 |
| Minimum and maximum number of MOTUs | 14 – 41 | 2-10 | 228 – 312 | 501 – 624 | 103 – 167 |
| Mean number of  MOTUs | 27.31 | 3.54 | 275.41 | 555.00 | 130.33 |
