# Supplementary material for: A DNA barcode library of Austrian geometridae (Lepidoptera) reveals high potential for DNA-based species identification
Source: PLoS One. 2024 Mar 11;19(3):e0298025. doi: 10.1371/journal.pone.0298025 (PMC10927147; doi:10.1371/journal.pone.0298025)
Supplement: S1 Text — (DOCX) [file pone.0298025.s003.docx]

Genbank accession numbers of sequences in Schattanek-Wiesmair et al., “A DNA barcode library of Austrian Geometridae (Lepidoptera) reveals high potential for DNA-based species identification”

FJ581449-FJ581450, FJ581454-FJ581455, GU655467-GU655468, GU655809, GU686763, GU686768, GU686774, GU686789, GU686806, GU686820, GU686822, GU687166-GU687167, GU687171, GU687173, HM376817, HM376825-HM376826, HM376828, HM381351-HM381352, HM381356, HM392668, HM392671, HM392675, HM392691, HM392695, HM392698, HM392701, HM392703-HM392704, HM392709, HM392801, HM392803, HM392805, HM392810, HM392812-HM392813, HM392815-HM392818, HM392831, HM392833-HM392840, HM392849-HM392850, HM393957, HM393962-HM393963, HM393968, HM394032-HM394035, HM394039, HM394043-HM394045, HM394213, HM425743-HM425754, HM425757, HM425763-HM425770, HM425775, HM425777, HM425782, HM425788, HM425796, HM425798, HM425800-HM425801, HM425806-HM425807, HM425810, HM425857-HM425860, HM425863, HM425865, HM425869-HM425871, HM425957, HM425981, HM872300, HM902911-HM902912, HM903217, HM903298-HM903307, HM903328, HM910656, HM910667, HQ564928, HQ957541, HQ957544, HQ957546, HQ957548, HQ957864, HQ957952, HQ958261, HQ968173-HQ968174, HQ968307, HQ968370, HQ968376, HQ968492, HQ968499, HQ968679-HQ968680, JF851430-JF851431, JF851434, JF860082, JF860293-JF860294, JF860306-JF860309, JF860312-JF860313, JF860339, JF860369, JN267821-JN267825, JN267830, JN267832, JN269334-JN269348, JN274054-JN274060, JN279533-JN279534, JN279558-JN279575, JN285757, JQ616293, JQ616297, JQ616308-JQ616309, JQ616380, JQ616385, JQ616502, JQ616528-JQ616529, JQ616690, JQ616720, JQ616726, JX34572, JX34592, JX34601, JX34604, JX34635, JX34638, JX34645, JX34671, JX34673-JX34674, KF807158, KF807175, KF807214, KF807263, KF807267, KF807271, KF807279, KF807302, KF807374, KF807390, KF807436, KF807538, KF807555, KF807655, KF807657, KF807688, KF807763, KF807766, KF807782, KF807801, KF807896, KF807911, KF808013, KF808137, KM571962, KM571965, KM572002, KM572009, KM572017, KM572019, KM572040, KM572062, KM572072-KM572073, KM572076, KM572089, KM572091, KM572093, KM572109, KM572127, KM572134, KM572145, KM572160, KM572163, KM572171-KM572172, KM572181, KM572187, KM572215, KM572222, KM572224, KM572243-KM572244, KM572267, KM572273, KM572284, KM572292, KM572298, KM572301, KM572306, KM572315, KM572318, KM572325, KM572341, KM572390, KM572393, KM572395, KM572401, KM572414, KM572417, KM572419, KM572423-KM572424, KM572433, KM572442, KM572447, KM572449, KM572452, KM572454, KM572505, KM572537, KM572542, KM572561, KM572563, KM572575, KM572578, KM572582, KM572619, KM572621-KM572622, KM572625, KM572655, KM572660, KM572696, KM572709, KM572717, KM572730, KM572735-KM572737, KM572749-KM572750, KM572782, KM572787, KM572799-KM572800, KM572809, KM572815, KM572829, KM572844, KM572850, KM572867, KM572878, KM572881, KM572883, KM572911, KM572917-KM572918, KM572932, KM572939, KM572954, KM572963, KM572975, KM572981, KM573042, KM573045-KM573046, KM573051, KM573057, KM573060, KM573087, KM573104, KM573121, KM573131, KM573134, KM573158, KM573171-KM573172, KM573191, KM573237, KM573242, KM573257, KM573286-KM573287, KM573296, KM573301, KM573374-KM573375, KM573381-KM573382, KM573389, KM573400, KM573407-KM573408, KM573412, KM573414, KM573422, KM573431, KM573442, KM573460, KM573465, KM573467, KM573476-KM573478, KM573482, KM573487, KM573498, KM573504, KM573517, KM573532-KM573533, KM573537, KM573559, KM573561, KM573570, KM573573, KM573578, KM573584, KM573586-KM573587, KM573589, KM573605, KM573612, KM573615, KM573624, KM573669, KM573688, KM573691, KM573702, KM573707, KP150209, KP253127, KP253137, KP253144, KP253146, KP253162, KP253168, KP253172, KP253176, KP253186, KP253194, KP253196, KP253198, KP253202, KP253205, KP253225, KP253243, KP253250, KP253253, KP253263, KP253269-KP253270, KP253279, KP253304, KP253316, KP253322, KP253342, KP253345, KP253347, KP253350, KP253355, KP253359, KP253368, KP253375, KP253395, KP253398, KP253405, KP253414-KP253415, KP253418, KP253428, KP253431, KP253436, KP253441, KP253450, KP253452, KP253456-KP253457, KP253461, KP253479, KP253481, KP253486, KP253501, KP253511, KP253521-KP253522, KP253533, KP253540, KP253588, KP253592, KP253595, KP253599, KP253601, KP253611, KP253615, KP253618, KP253638, KP253650, KP253663-KP253664, KP253674, KP253689, KP253694, KP253711, KP253730, KP253745, KT782348, KX17216, KX40304, KX40889, KX41036, KX41982, KX41998, KX42010, KX42028, KX42047, KX42060, KX42125, KX42145, KX42167, KX42222, KX42243, KX42340, KX42353, KX42384, KX42479, KX42483, KX42549, KX42583, KX42591, KX42608-KX42609, KX42614, KX42643, KX42677, KX42706, KX42822, KX44550, KX44611, KX44974, KX45269, KX45428, KX45552, KX45572, KX45601, KX45610, KX45703, KX45764, KX45783, KX46069, KX46377, KX46392, KX46551, KX46689, KX46743, KX46803, KX46892, KX46945, KX47101, KX47164, KX47327, KX47354, KX47423, KX47479, KX47543, KX47576, KX49611, KX70778, KX70856, KX70878, KX70899, KX70901, KX70961, KX70963, KX70986, KX70988, KX71014, KX71052, KX71073, KX71137, KX71163, KX71169, KX71213, KX71239, KX71270, KX71274, KX71305, KX71322, KX71351, KX71357, KX71364, KX71367, KX71385, KX71390, KX71395, KX71397, KX71428, KX71430, KX71470, KX71483, KX71509, KX71544, KX71571, KX71597, KX71618, KX71669, KX71681, KX71684, KX71775, KX71791, KX71800, KX71838, KX71840, KX71843, KX71868, KX71911, KX71927, KX71938, KX71945, KX71977, KX71979, KX72071, KX72087, KX72094, KX72134, MG521914, MG521931-MG521932, MG521944, MG521990, MG521994, MG522008, MG522018, MG522041, MG522066, MG522125, MG522127, MG522139, MG522145-MG522146, MG522202, MG522236, MG522239, MG522262, MG522285, MG522297, MG522318, MG522391, MG522449, MG522481, MG522485, MG522491, MG522496, MG522543, MG522547, MG522578, MG522614, MG522643, MG522698, MG522704, MG522718, MG522731, MG522733, MG522736, MG522760, MG522768, MG522775, MG522800, MG522810, MG522829, MG522831, MG522838, MG522841, OK623703-OK623704, OR367739-OR369720
