# Supplementary material for: A DNA barcode library of Austrian geometridae (Lepidoptera) reveals high potential for DNA-based species identification
Source: PLoS One. 2024 Mar 11;19(3):e0298025. doi: 10.1371/journal.pone.0298025 (PMC10927147; doi:10.1371/journal.pone.0298025)
Supplement: Attachment — Submitted filename: Comments.docx [file pone.0298025.s004.docx]

Dear Editor and authors,

I congratulate the authors established a DNA barcode reference library for all the species of Geometridae that have recorded in Austria, and updated the checklist of the

geometrid moths of Austria. The results of this study provides new and comprehensive species diversity resources of Geometridae. However, the writing of the article needs to be improve. The redundancy of sentences is the main problem of this manuscript. I advised the authors to read more articles of DNA barcode to polished the word used in this manuscript. You can also reference the revisions I listed as follows.

Minor comments:

L22: we report a complete DNA barcode library DNA of all the 476 22 species of Geometridae (Lepidoptera) that have been recorded in Austria.

L23: Austria. Species were

L24: capture the intraspecific genetic variation

L25: 2500 DNA barcode sequences

L27: with DNA barcodes from

L28: by ASAP, BIN and bPTP methods yielded

L31:whereas bPTP appeared to overestimate the number of species.

L33-L35: The potential of DNA barcode on species identification has been confirmed over a broad spectrum of biological groups, especially in Lepidoptera. You should clarify that your the DNA barcoding library combining xxx species delimitation method is potential for the species identification of Geometridae (if it is not reported before your study).

L60: Why do you only compared with the countries of Central European, but not Asia or America?

L61-L63: “species counts have been on the rise in recent decades” and “decline of overall Biomass” , contradiction?

L69:You should summarize how many species have been obtained the DNA barcode in the world and Austria, respectively. And how many species have not been obtained the DNA barcode.

L70: there is no right parenthesis, please check.

L81: the taxonomic work of which taxa group?

L104: The present study assembled 2500 DNA barcodes of all 476 geometrid species from Austrian, and captured a intraspecific genetic variation based on the broad geographic coverage sampling.

L115: we obtained DNA barcode sequences

L116: Herein, 438 species

L116:at least one specimen

L117:by the specimens from non-Austrian

L119: to this study

L119-L120: study, most specimens are originating from Tiroler

L124: We divided Austria in three

L113: the three areas

L132-L133: Do you mean sample at least three specimens per species in each defined area? Why the species were known to occur in only one or two of the defined areas could be sampled at least three specimens per species, the species were known to occur in three area should not?

L135-L138: we retrieved 38 COI sequences of these 38 species from the BOLD database, and their sampling sites are near Austria (mostly from Germany......)

L151: sequencing the DNA barcode region

L152:The DNA barcode sequences

L153:generated based on a standard high-throughput protocol [53] with primers

L154-156: LepF2 [54]. The length of sequences met the criterias of BOLD system (Barcode of Life Data System, www.boldsystems.org[55]; i.e., at least 500 bp) were retained for the subsequent analyses.

‘BOLD’ should be show as full name for the first appearance, and next time just show BOLD is ok. Please check that throughout the manuscript

L156: specimen collection data and

L157-158: Austria” in the BOLD, and DNA sequences were available in

L158-159:DNA sequences were also deposited in Genbank (Genbank accession numbers XXX -XXX)

L162: For each species, The the nearest neighbor distance and the maximum intraspecific distance were calculated by the Barcode Gap Analysis tool of the BOLD system, based on the Kimura two-parameter (K2P) model.

The meaning of “the nearest” is same to “ the minimum” for nearest neighbor distance.

L168-170: taxonomic assignment. The species with BIN split were checked for the morphological identification. These data

L175: “The assignment of species to subfamilies follows [30].” is redundancy, you have mentioned in L139. And the L139 should be polished.
