# Supplementary material for: A DNA barcode library of Austrian geometridae (Lepidoptera) reveals high potential for DNA-based species identification
Source: PLoS One. 2024 Mar 11;19(3):e0298025. doi: 10.1371/journal.pone.0298025 (PMC10927147; doi:10.1371/journal.pone.0298025)
Supplement: Attachment — Submitted filename: Comments _R2.docx [file pone.0298025.s006.docx]

Comments to the Author

The authors made efforts in improving the manuscript. And a few revisions are required. I congratulate the authors for this ambitious study. Revisions are as follows:

L25: In total, 2500 DNA barcode sequences (“DNA barcode” can be more precise, “barcode” could be barcode of goods. Some professors who study in DNA barcode have given me that suggestion. So please check that throughout the manuscript)

L137: “sites(mostly from” change to “sites (mostly from”

L162: calculated on the BOLD system

L184: MAFFT, Modelfinder and IQ-TREE should be clarified the version number, please check all software throughout the manuscript.

L204: “and 2525 sequences were assigned a Barcode Index Number (BIN)” Do you mean “unique” BIN? If not, why are the remaining 42 sequences not assigned BIN? Please clarify.

L220: 7.1 % of xxx. It is ambiguous. Please clarify.

L246: 27 morphospecies presented sharing BINs.(Your original sentence could be mean “27 morphospecies, they shared BINs with each other”)

L283: , and two BINs were presented in each of 47 species.

L309: Based on the ASAP method

L364-365: ASAP performed similarly well in morphological taxonomy, it is actually related to the DNA barcode gap (max. Intraspec and min.NN ), it would be better if you discuss about that.

L400: Please check that Fig 7 is right (the Fig number)

L430-432: Please show the relevant figure of genitalia.

L464: cf. should be followed by the species name. Please clarify.

For Table 1: as you show in Table 1, most of species sharing BINs show differentiation in wing coloration and wing shape. If that suggested the wing coloration and wing shape may be not suitable for species identification? If there are some controversy on using the wing coloration and wing shape to identify species? You should discuss that in Discussion.

L278: Fig5: You should clarify the tree you have showed, for example, NJ tree or ML tree. The figure and its legend must be understandable without reference to the text.

And the species name in Fig5 should be italic.

Please check the Fig7, Fig8, and Fig10, accordingly.

For Fig9: Please show the regression equation, R-value and P-value of the curve in the figure.
