# Supplementary material for: A DNA barcode library of Austrian geometridae (Lepidoptera) reveals high potential for DNA-based species identification
Source: PLoS One. 2024 Mar 11;19(3):e0298025. doi: 10.1371/journal.pone.0298025 (PMC10927147; doi:10.1371/journal.pone.0298025)
Supplement: Attachment — Submitted filename: Rresponse to Reviewers_formatted.docx [file pone.0298025.s007.docx]

Dear Editors,

In the following, we respond to each of the referee’s comments and explain the changes made in the manuscript. Reviewer comments are in bold print.

**Comments to the Author**

**The authors made efforts in improving the manuscript. And a few revisions are required. I congratulate the authors for this ambitious study. Revisions are as follows:**

**L25: In total, 2500 DNA barcode sequences (“DNA barcode” can be more precise, “barcode” could be barcode of goods. Some professors who study in DNA barcode have given me that suggestion. So please check that throughout the manuscript)**

REPLY: We checked all occurrences of “barcode” and replaced them with “DNA barcode”.

**L137: “sites(mostly from” change to “sites (mostly from”**

REPLY: we inserted the space

**L162: calculated on the BOLD system**

REPLY: changed as suggested by the reviewer

**L184: MAFFT, Modelfinder and IQ-TREE should be clarified the version number, please check all software throughout the manuscript.**

REPLY: we added version number to all software, provided that version numbers are attached to the software (this was not the case for ASAP and bPTP)

**L204: “and 2525 sequences were assigned a Barcode Index Number (BIN)” Do you mean “unique” BIN? If not, why are the remaining 42 sequences not assigned BIN? Please clarify.**

REPLY: No, we do not mean “unique BIN”; these are sequences that were assigned a BIN number, which could be shared with other sequences. There are several possible reasons for why some (i.e., 42) sequences were not assigned a BIN at the time of data analysis – for instance that sequences were too short, references flagged as false identification, or only recently uploaded.

**L220: 7.1 % of xxx. It is ambiguous. Please clarify.**

REPLY: was changed to “7.1 % of 476 species”

**L246: 27 morphospecies presented sharing BINs.(Your original sentence could be mean “27 morphospecies, they shared BINs with each other”)**

REPLY: we changed the wording to “27 morphospecies shared BINs with another morphospecies”

**L283: , and two BINs were presented in each of 47 species.**

REPLY: We changed the wording to “In Austria, 91 of these species are represented with a single BIN each, and 47 species are each represented with two BINs (Fig 6; S1 Table)”.

**L309: Based on the ASAP method**

REPLY: changed as suggested by the reviewer

**L364-365: ASAP performed similarly well in morphological taxonomy, it is actually related to the DNA barcode gap (max. Intraspec and min.NN ), it would be better if you discuss about that.**

REPLY: we revised the sentence: “In the present study, BIN and ASAP performed similarly well in terms of congruence between reconstructed MOTUs and morphological taxonomy”.

All three algorithms seek to identify the point, where intraspecific diversity can be distinguished from interspecific diversity, so the barcode gap is relevant for each of the methods.

**L400: Please check that Fig 7 is right (the Fig number)**

REPLY: the figure number was corrected.

**L430-432: Please show the relevant figure of genitalia.**

REPLY: we include photographs of genitalia of male and female

**L464: cf. should be followed by the species name. Please clarify.**

REPLY: the “cf” was a typo and deleted

**For Table 1: as you show in Table 1, most of species sharing BINs show differentiation in wing coloration and wing shape. If that suggested the wing coloration and wing shape may be not suitable for species identification? If there are some controversy on using the wing coloration and wing shape to identify species? You should discuss that in Discussion.**

REPLY: When BIN sharing species differ in morphological traits, BIN sharing can be a consequence of introgression, i.e. hybridization followed by introduction of one species’ mt DNA haplotype into the other species. These findings do not raise a controversy particularly on the use of wing coloration or wing shape for species identification, as the phenomenon can concern any morphological trait.

**L278: Fig5: You should clarify the tree you have showed, for example, NJ tree or ML tree. The figure and its legend must be understandable without reference to the text.**

REPLY: we added the method (Neighbor joining) to the figure legend

**And the species name in Fig5 should be italic.**

**Please check the Fig7, Fig8, and Fig10, accordingly.**

REPLY: changed as requested in all figure legends

**For Fig9: Please show the regression equation, R-value and P-value of the curve in the figure.**

REPLY: The line in the graph shows the predictions of a negative binomial regression model. The relationship between the predictor and the response variable in the regression equation is logarithmic. Therefore, the regression equation cannot easily be interpreted and would not add useful information to the graph. To comply with the reviewer’s request to report the equation, we included it in the figure description. We do not repeat the p-value in the figure or its description, as it is reported in the text together with the effect size estimate (effect size, test statistic and p-value are reported as est. = 0.08, z = 8.78, p < 10^-16^).
